# Supplementary figures and images for: Mobilization of nuclear antiviral factors by exportin XPO1 via the actin network inhibits RNA virus replication
Source: PLoS Pathog. 2025 Aug 19;21(8):e1012841. doi: 10.1371/journal.ppat.1012841 (PMC12393752; doi:10.1371/journal.ppat.1012841)

**S1 FIG**

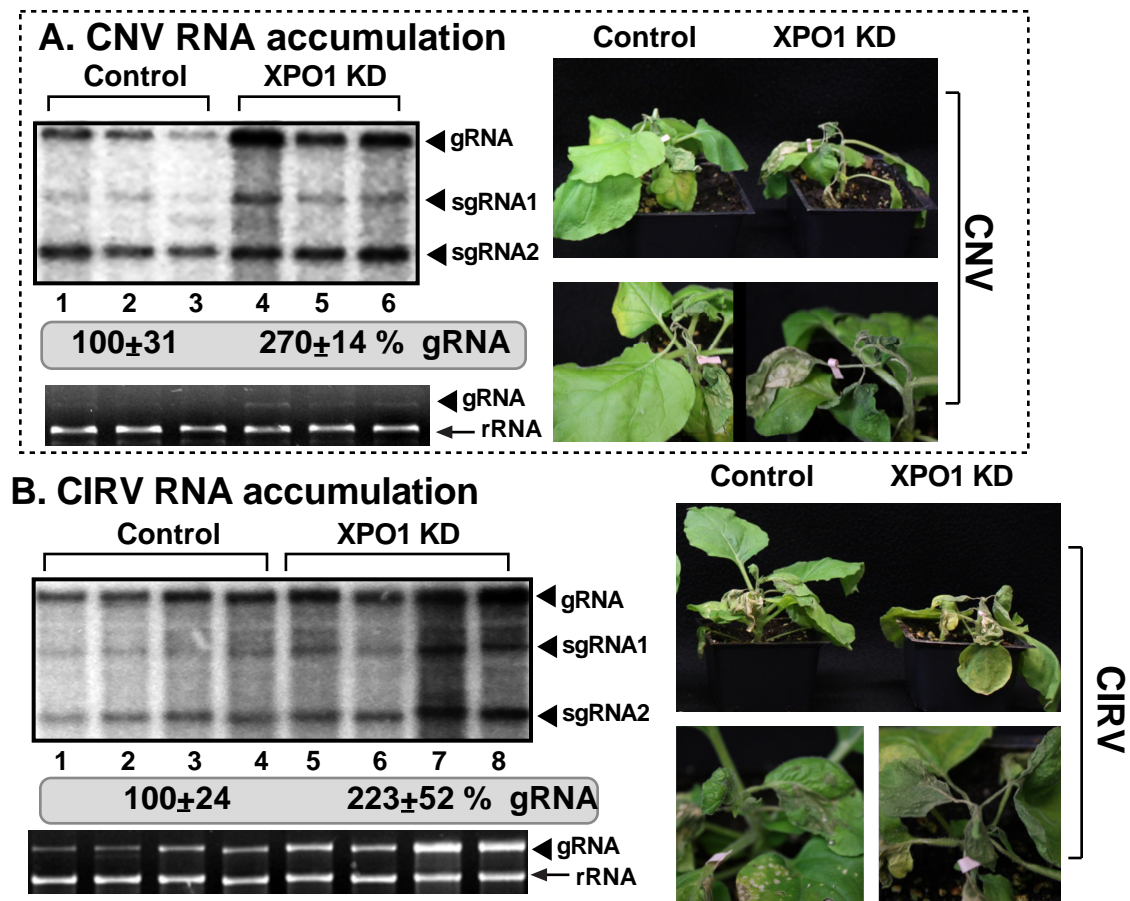

Supplement: S1 Fig — (A) Accumulation of CNV genomic (g)RNA and subgenomic (sg)RNAs at 2 days after CNV inoculation in XPO1-silenced (KD) plants was measured by northern blot analysis. The inoculation with CNV was performed 7 days after VIGS. Second panel: The ribosomal RNA is shown as the loading control in agarose gel stained with ethidium-bromide. CNV gRNA is visible in the gel. Right panel: Accelerated and more severe CNV-induced symptom development was observed in XPO1-silenced N. benthamiana. The symptoms were documented at 5 days post CNV inoculation. (B) Left panels: Accumulation of CIRV gRNA in XPO1-silenced N. benthamiana plants at 2 d post inoculation (dpi) was measured by northern blot analysis. See further details in panel A. Each experiment was repeated three times. (PDF) [file ppat.1012841.s001.pdf]

S2 FIG

A. CNV RNA accumulation

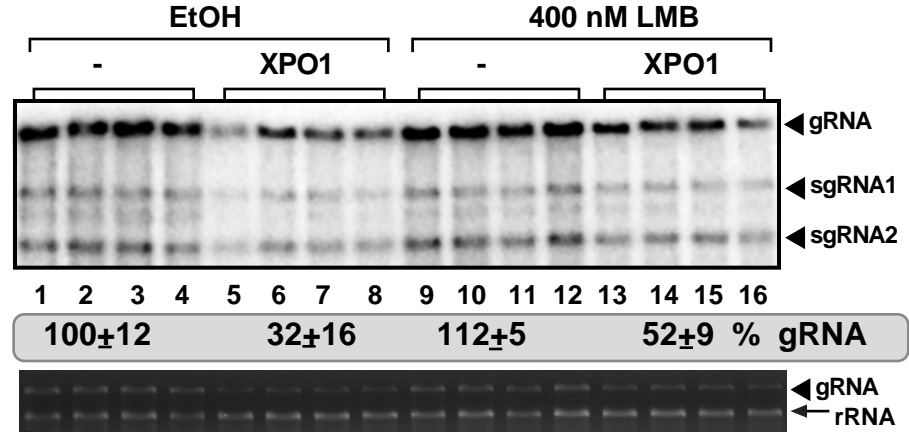

B. CIRV RNA accumulation

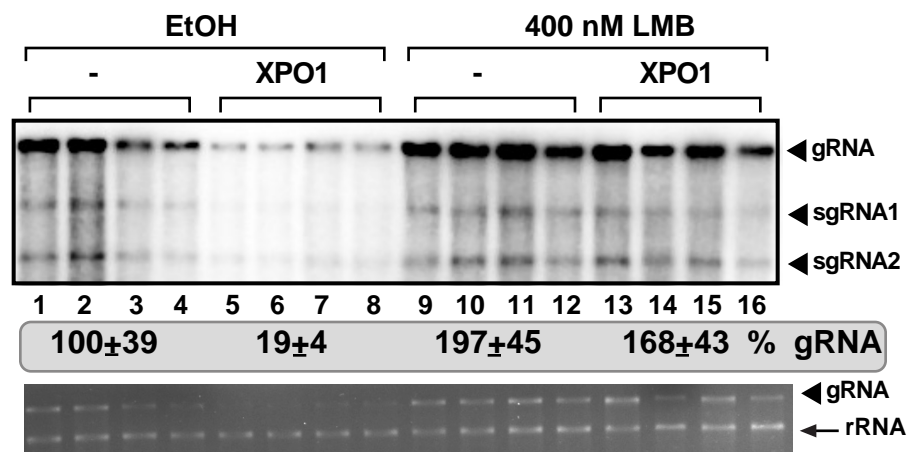

C

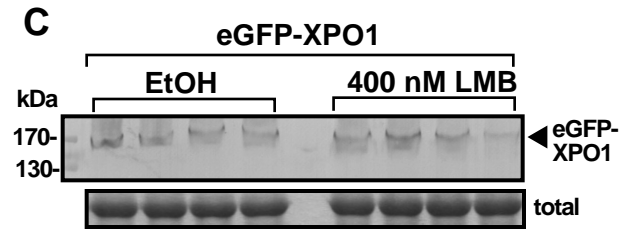

Supplement: S2 Fig — (A-B). Transient expression of XPO1 in N. benthamiana inhibits accumulation of CNV and CIRV, respectively. Top panels: Accumulation of CNV or CIRV RNAs at 2 dpi was measured by northern blot analysis. Bottom panel: The 18S ribosomal RNA is shown in an agarose gel stained with ethidium-bromide as the loading control. N. benthamiana leaves were agroinfiltrated for transient expression of XPO1 (pGD vector as control), in combination with 0.5% ethanol (EtOH) as a control or 400 nM of Leptomycin B (LMB), a chemical inhibitor of XPO1. The LMB and EtOH treatments were repeated 24 h later. (C) Expression of XPO1 was measured by western blot using anti-GFP antibody. Coomassie Brilliant Blue staining was used for the normalization of total proteins as loading control. (PDF) [file ppat.1012841.s002.pdf]

**A. Co-localization / CIRV**

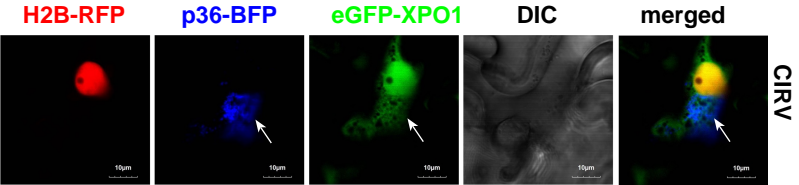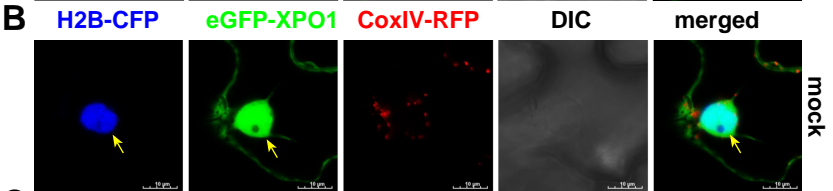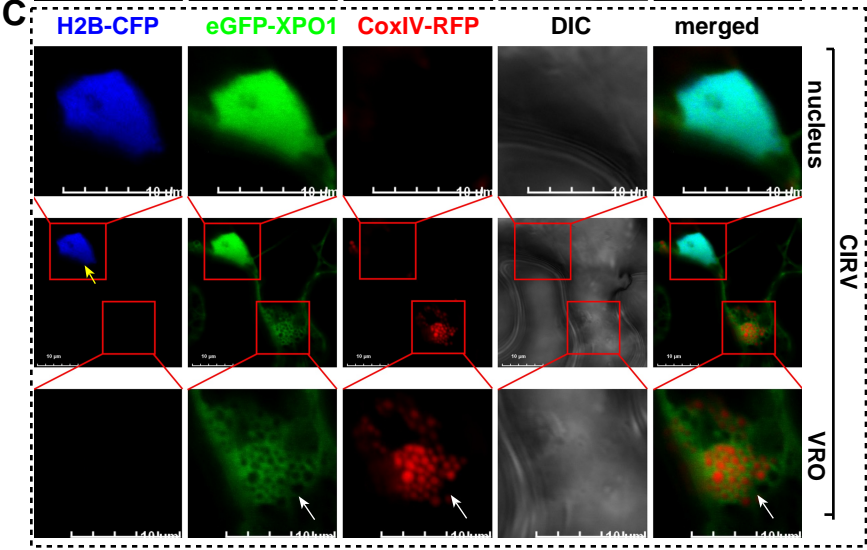

**D. Co-localization / CNV**

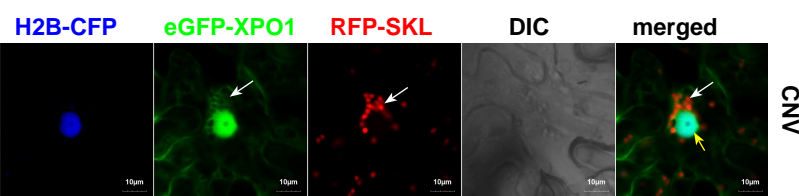

Supplement: S3 Fig — (A) Confocal laser microscopy images show co-localization of CIRV p36-BFP replication protein and eGFP-XPO1 during CIRV replication. Transgenic N. benthamiana expressing H2B-RFP as the nuclear marker was used. Scale bars represent 10 μm. (B) The subcellular localization of eGFP-XPO1 in the mock-treated N. benthamiana. Mitochondria are indicated by CoxIV-RFP. Scale bars represent 10 μm. (C) Note that eGFP-XPO1 partitioned between the nucleus and VROs during CIRV replication. Middle panel: The nuclear localization and relocation to VROs of eGFP-XPO1 were captured in the same plant cell. Top panel: Enlarged images of the nuclear area. Bottom panel: Enlarged images of the VRO region. Scale bars represent 10 μm. (D) Confocal laser microscopy images show partial re-localization of eGFP-XPO1 into CNV VROs decorated by RFP-SKL marker. The VRO is pointed at by a white arrow, whereas the nucleus is marked by a yellow arrow. Transgenic N. benthamiana expressing H2B-CFP as the nuclear marker was used in (B, C and D). Scale bars represent 10 μm. Each experiment was repeated three times. (PDF) [file ppat.1012841.s003.pdf]

S4 FIG

A. BiFC

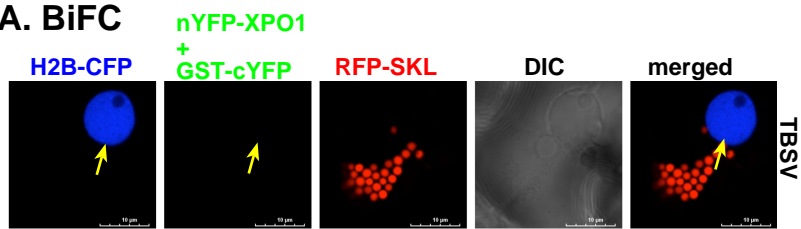

B

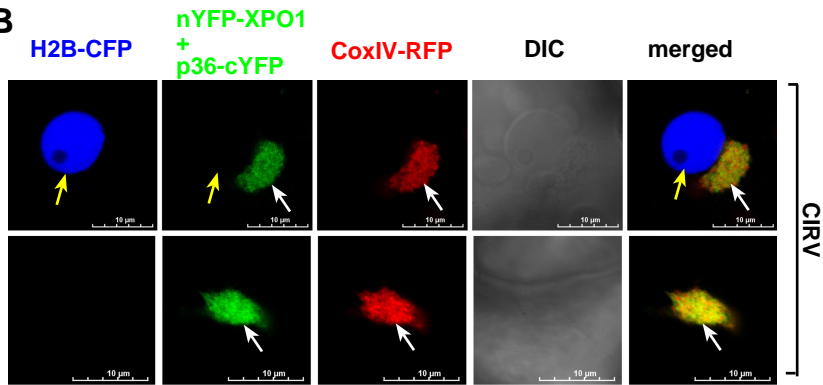

C. CIRV

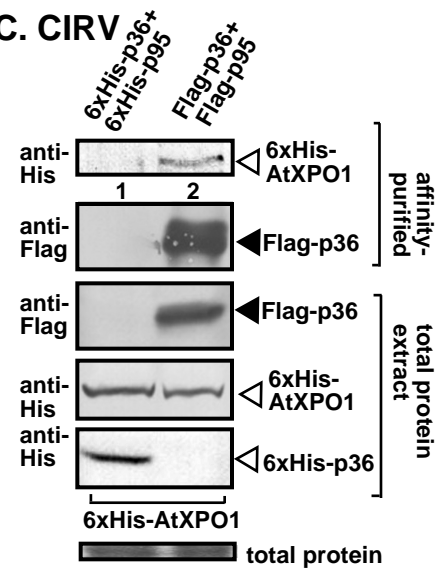

Supplement: S4 Fig — (A) Control BiFC experiments with the combination of GST-cYFP and nYFP-XPO1 was performed in H2B-CFP transgenic N. benthamiana infected with TBSV. The nucleus is marked by a yellow arrow (B) Interaction between CIRV p36-cYFP and nYFP-XPO1 was identified by BiFC during CIRV replication in H2B-CFP transgenic N. benthamiana. The VROs, consisting of clustered mitochondria, are visualized by CoxIV-RFP mitochondrial marker. The interaction signals in VROs are indicated by white arrows, whereas the nucleus is marked by a yellow arrow. Scale bars represent 10 μm. (C) Copurification of CIRV Flag-p36 and Flag-p95pol replication proteins with His6-XPO1 from subcellular membranes of yeast. Top two panels: Co-purified His6-XPO1 (lane 2) and Flag-affinity-purified Flag-p36 were detected using western blot analysis. The negative control yeasts expressed His6-p36 and His6-p95pol together with His6-XPO1. Middle three panels: Identification of protein expression in total samples from yeasts were detected by western blotting using the shown antibodies. Each experiment was repeated three times. (PDF) [file ppat.1012841.s004.pdf]

S5 FIG

A. BiFC

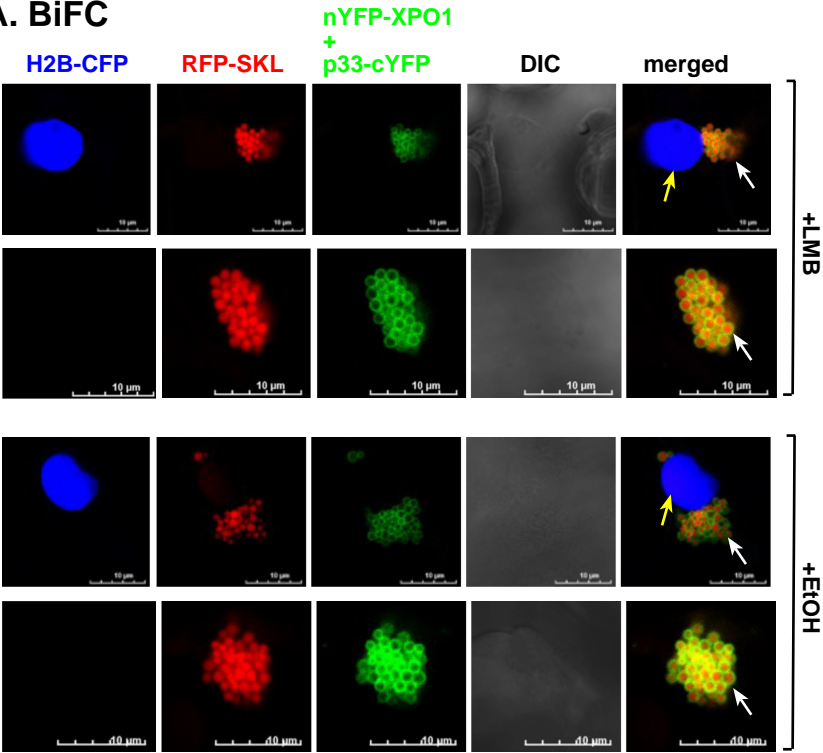

B. BiFC

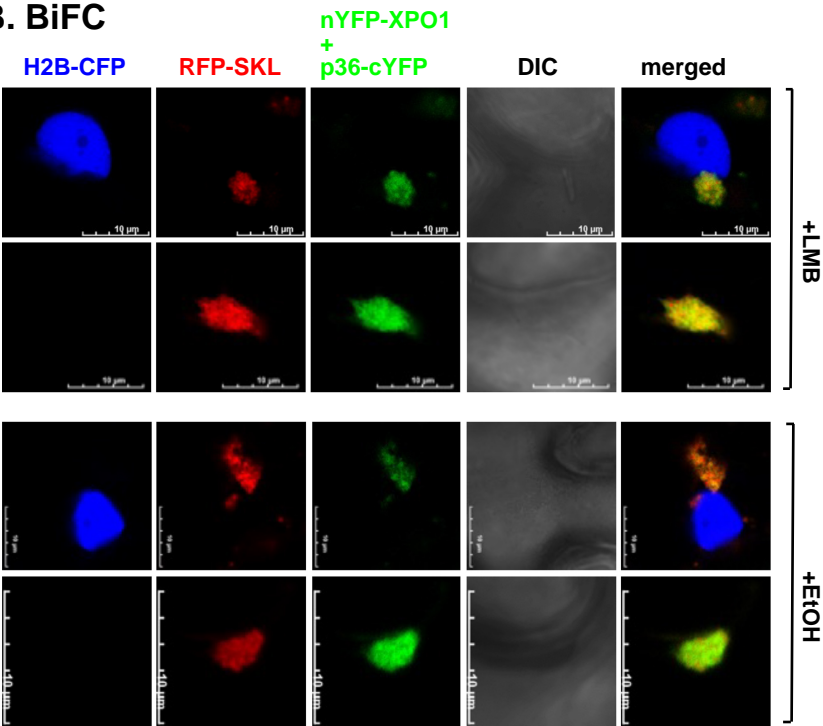

Supplement: S5 Fig — (A-B) Interaction between TBSV p33-cYFP or CIRV p36-cYFP and nYFP-XPO1 was identified by BiFC in H2B-CFP transgenic N. benthamiana. The leaves were treated with either 400 nM LMB or 0.5% EtOH (control) twice via infiltration. The VROs, consisting of either clustered peroxisomes or clustered mitochondria, are visualized by either RFP-SKL or CoxIV-RFP mitochondrial marker. We show two sets of images to demonstrate that VROs localize either proximal to nucleus (top images) or, more frequently, at distal positions (bottom images). The interaction signals in VROs are indicated by white arrows, whereas the nucleus is marked by a yellow arrow. (PDF) [file ppat.1012841.s005.pdf]

S6 FIG

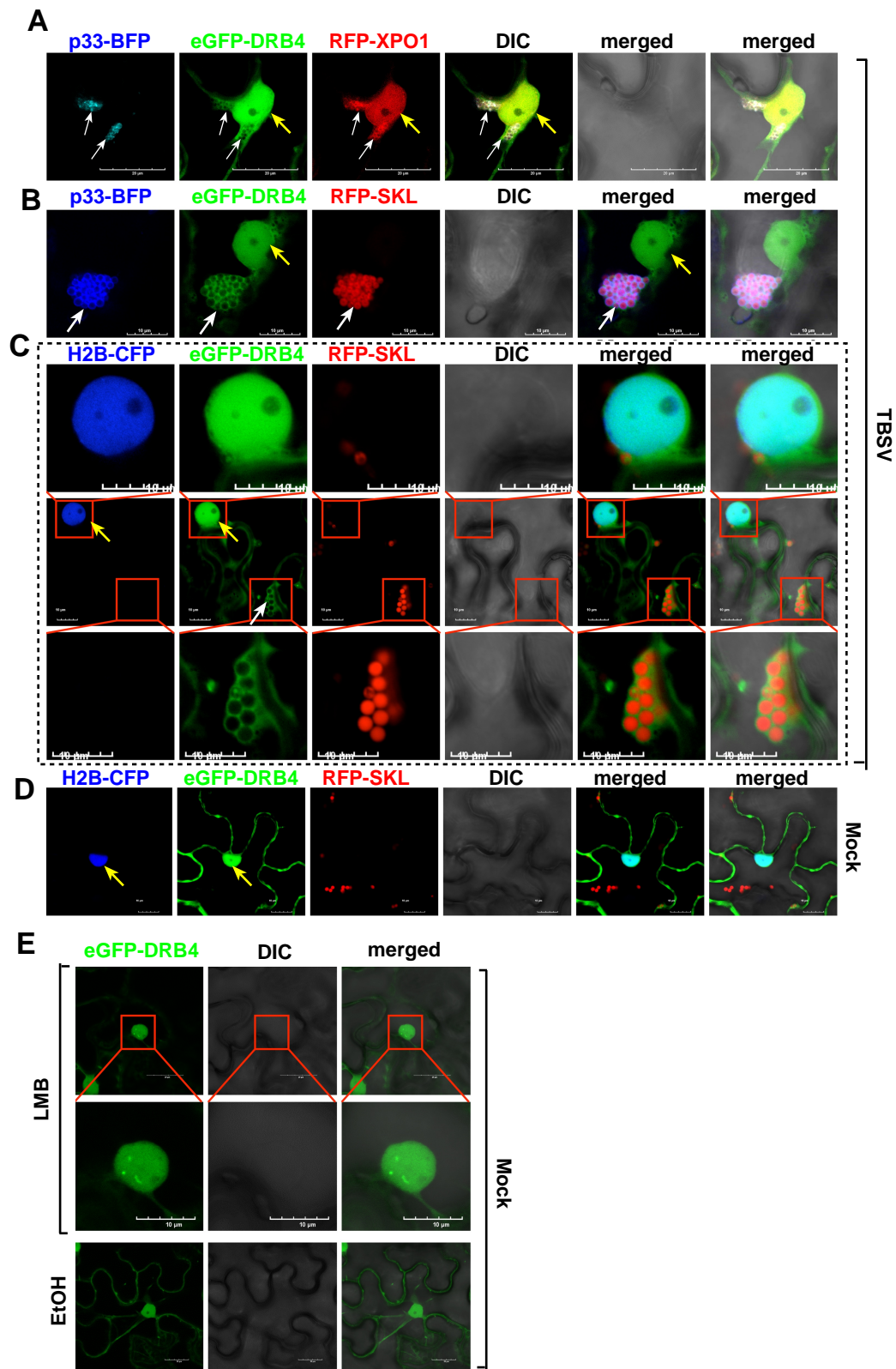

Supplement: S6 Fig — (A) Confocal microscopy images show colocalization of eGFP-DRB4 and RFP-XPO1 in VROs marked with p33-BFP replication protein during TBSV replication in N. benthamiana. Scale bars represent 10 μm. (B) A fraction of eGFP-DRB4 is relocated to VRO marked by p33-BFP, which includes clustered peroxisomes (marked with by RFP-SKL) during TBSV replication in N. benthamiana. The nucleus is pointed at by a yellow arrow. Note that the VRO is localized at proximal position to nucleus. Scale bars represent 10 μm. (C) Top panel: The enlarged images (the red boxed area in the middle panel) show partial nuclear localization of eGFP-DRB4 during TBSV replication. Middle panel: Confocal images of a single cell from a H2B-CFP transgenic N. benthamiana infected with TBSV. Note that the VRO is localized at distal position from the nucleus. Bottom panel: The enlarged images (the red boxed area in the middle panel) show eGFP-DRB4 is present in a single VRO during TBSV replication. Scale bars represent 10 μm. (D) Confocal microscopy images show the subcellular localization of eGFP-DRB4 in healthy plant cells of H2B-CFP transgenic N. benthamiana. (E) LMB chemical inhibitor of XPO1 restricts eGFP-DRB4 shuttle protein mostly in the nucleus. Top panels: eGFP-DRB4 is restricted in the nucleus when 400 nM LMB is co-infiltrated with the agrobacterium carrying pGD-eGFP-DRB4 in the healthy leaves of N. benthamiana. Bottom panel: eGFP-DRB4 localizes in the nucleus and cytosol when cells were treated with 0.5% EtOH as the control. Scale bars represent 10 μm. Each experiment was repeated three times. (PDF) [file ppat.1012841.s006.pdf]

S7 FIG

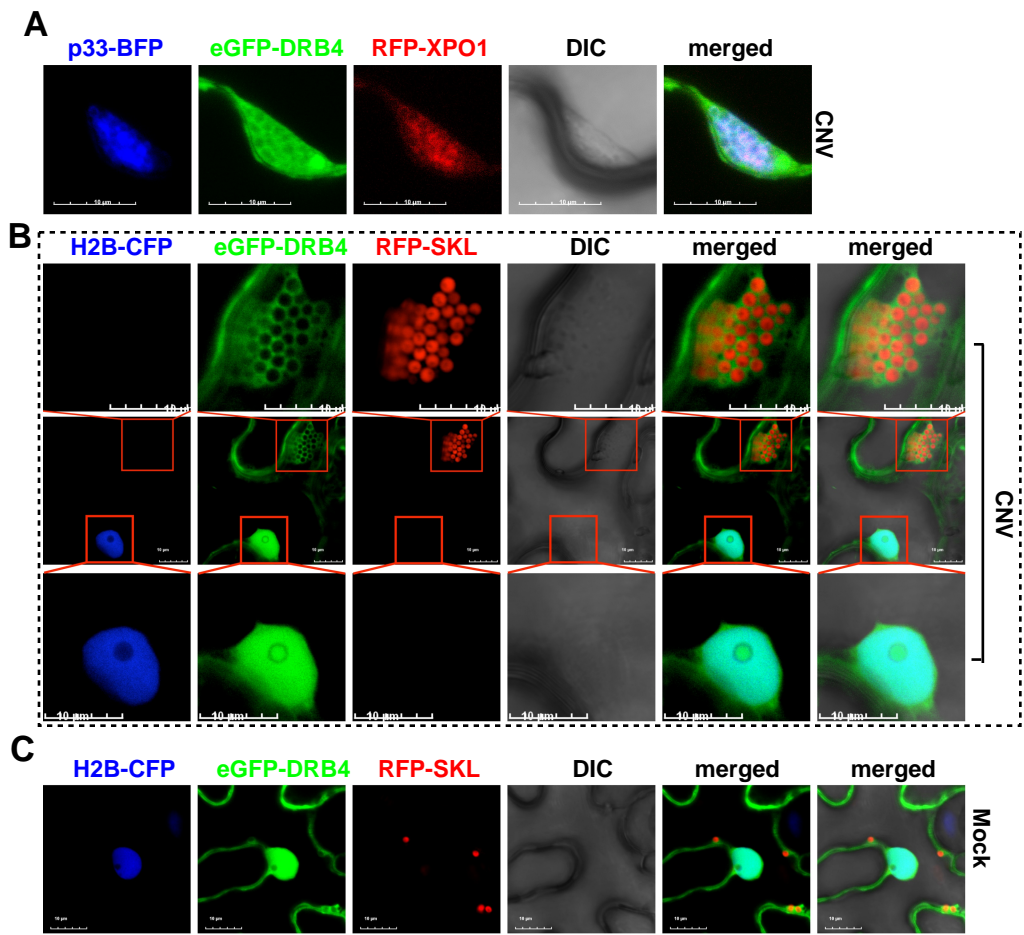

Supplement: S7 Fig — (A) Confocal microscopy images show colocalization of eGFP-DRB4 and RFP-XPO1 in VROs marked with p33-BFP replication protein during CNV replication in N. benthamiana. Scale bars represent 10 μm. (B) A fraction of eGFP-DRB4 is relocated to VROs represented by clustered peroxisomes (marked with by RFP-SKL) during CNV replication in H2B-CFP transgenic N. benthamiana. H2B-CFP is used as nuclear marker. Top panel: The enlarged images (the red boxed area in the middle panel) show eGFP-DRB4 is present in a single VRO during CNV replication. Middle panel: Confocal images of a single cell from a H2B-CFP transgenic N. benthamiana infected with CNV. Bottom panel: The enlarged images (the red boxed area in the middle panel) show partial nuclear localization of eGFP-DRB4 during CNV replication. Scale bars represent 10 μm. (C) Confocal microscopy images show the subcellular localization of eGFP-DRB4 in healthy plant cells of H2B-CFP transgenic N. benthamiana. Each experiment was repeated three times. (PDF) [file ppat.1012841.s007.pdf]

**S8 FIG**

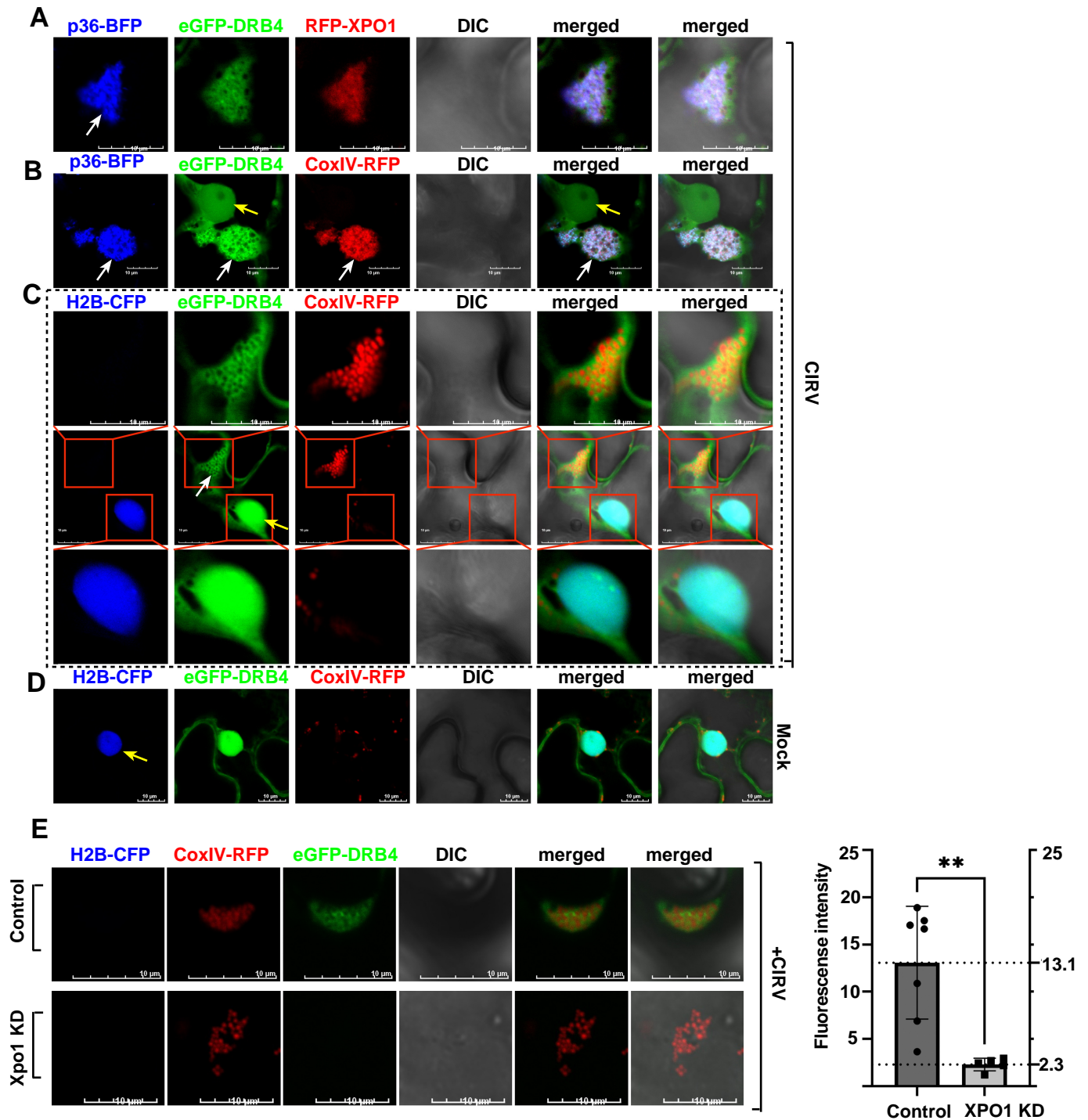

Supplement: S8 Fig — (A-D) Confocal microscopy images show localization of eGFP-DRB4 in VROs in H2B-CFP transgenic N. benthamiana infected with CIRV. CoxIV-RFP marks the clustered mitochondria representing CIRV VROs. Yellow arrows indicate the nucleus, while VROs are marked with white arrows. See further details in S6 Fig. (E) Confocal microscopic images show poor recruitment of eGFP-DBR4 into VROs (indicated by CoxIV-RFP) in XPO1 KD N. benthamiana infected with CIRV. Control experiment with N. benthamiana plants agroinfiltrated with the TRV vector carrying partial GST sequence. Scale bars represent 10 μm. Right panel: Quantification of fluorescent intensity of eGFP-DRB4 in CIRV VRO regions with Olympus FV3000 FLUO-view software. T-test is used for data analysis utilizing GraphPad Prism 9 (** represents P < 0.01). (PDF) [file ppat.1012841.s008.pdf]

S9 FIG

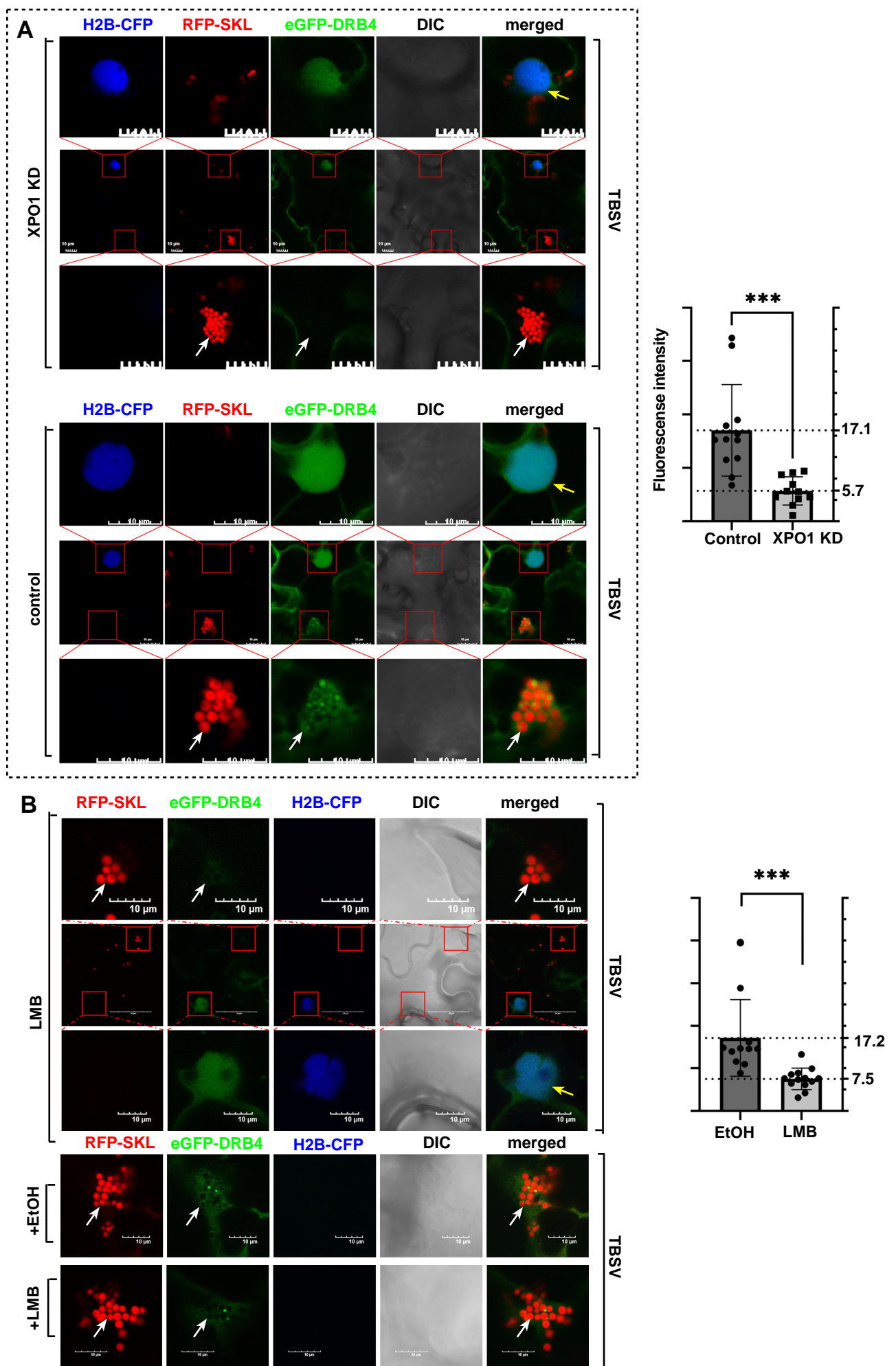

Supplement: S9 Fig — (A) Confocal microscopy images show re-localization of eGFP-DRB4 in VROs in H2B-CFP transgenic N. benthamiana infected with TBSV. XPO1 level was reduced by VIGS, whereas XPO1 was not targeted in control plants. RFP-SKL marks the clustered peroxisomes representing TBSV VROs, pointed at by white arrows, whereas the nucleus is pointed at by yellow arrow. Right panel: Quantification of fluorescent intensity of eGFP-DRB4 is shown in TBSV VRO regions in XPO1 KD leaves versus in controls. T-test is used for data analysis utilizing GraphPad Prism 9 (*** represents P < 0.001). See further details in S6 Fig. (B) Confocal microscopic images show poor recruitment of eGFP-DBR4 into VROs in 400 nM LMB-treated N. benthamiana infected with TBSV. Control experiment included 0.5% EtOH treatment of N. benthamiana leaves infected with TBSV. Scale bars represent 10 μm. Right panel: Quantification of fluorescent intensity of eGFP-DRB4 is shown in VRO regions in LMB-treated leaves versus in controls. (PDF) [file ppat.1012841.s009.pdf]

S10 FIG

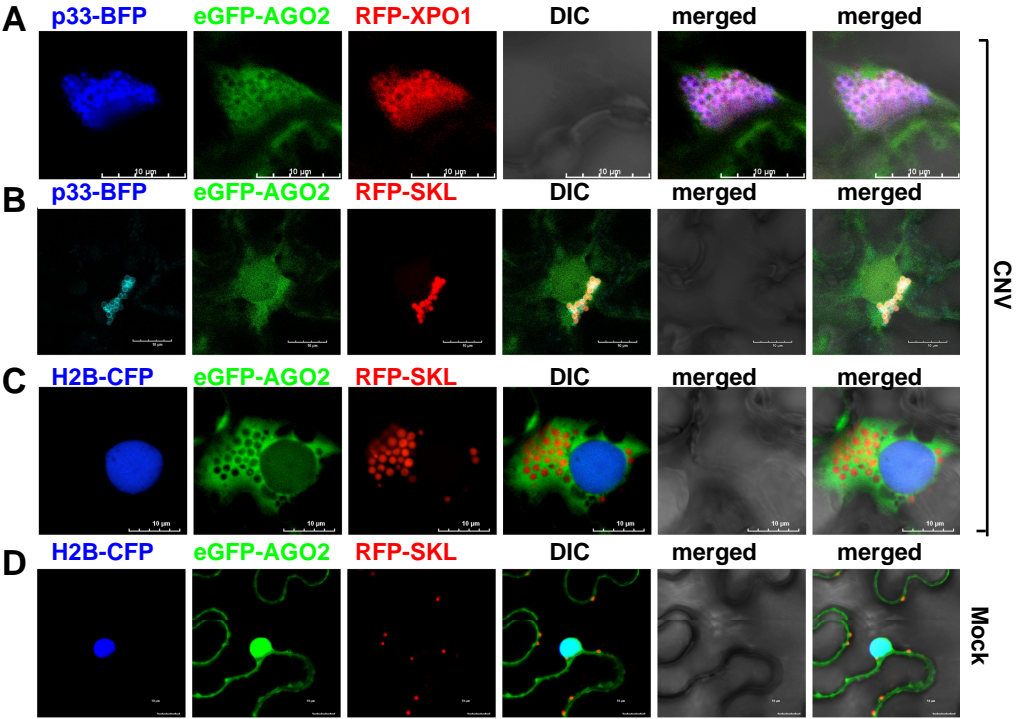

Supplement: S10 Fig — (A-D) Confocal microscopy images show partial re-localization of eGFP-AGO2 in VROs in H2B-CFP transgenic N. benthamiana infected with CNV or mock treated. See further details in S7 Fig. (PDF) [file ppat.1012841.s010.pdf]

S11 FIG

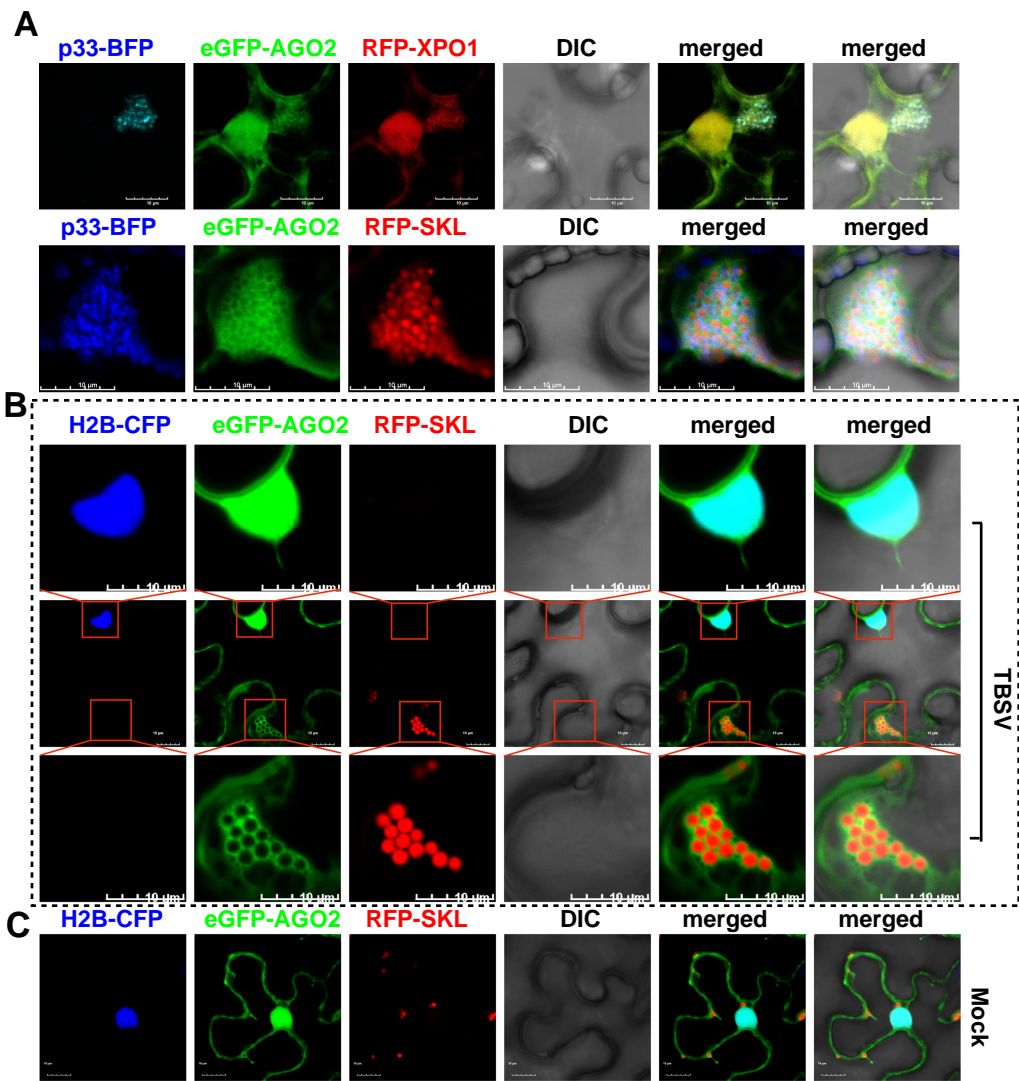

Supplement: S11 Fig — (A-C) Confocal microscopy images show partial re-localization of eGFP-AGO2 in VROs in H2B-CFP transgenic N. benthamiana infected with TBSV or mock treated. See further details in S6 Fig. (PDF) [file ppat.1012841.s011.pdf]

**S12 FIG**

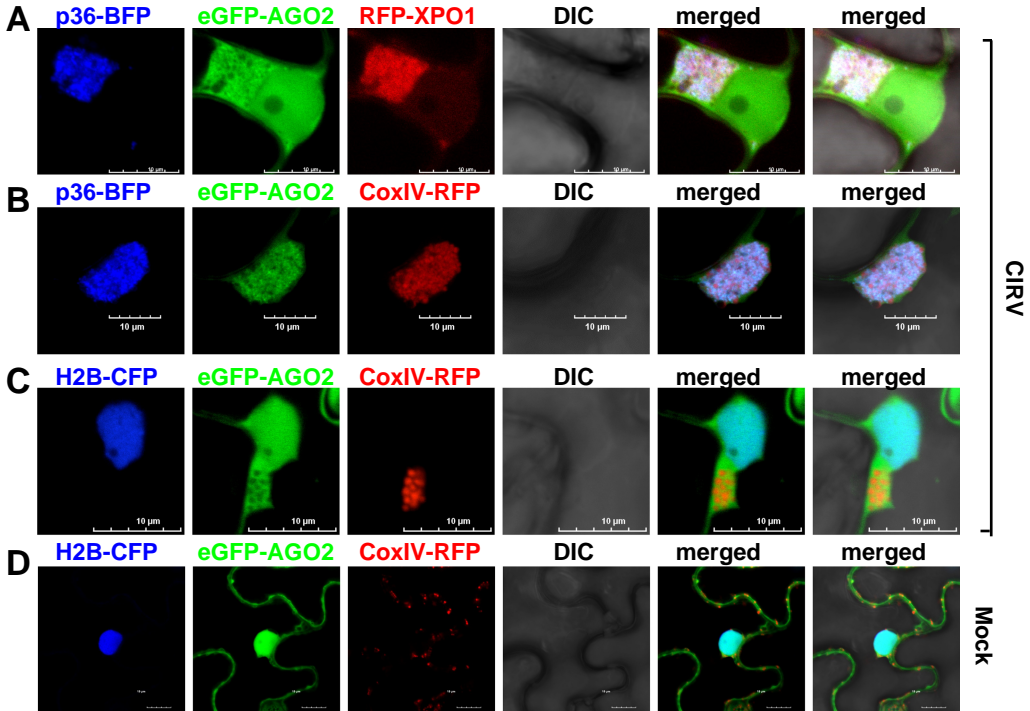

Supplement: S12 Fig — (A-D) Confocal microscopy images show partial re-localization of eGFP-AGO2 in VROs in H2B-CFP transgenic N. benthamiana infected with CIRV or mock treated. CoxIV-RFP marks the clustered mitochondria representing CIRV VROs. Scale bars represent 10 μm. See further details in S6 Fig. (PDF) [file ppat.1012841.s012.pdf]

# S13 FIG

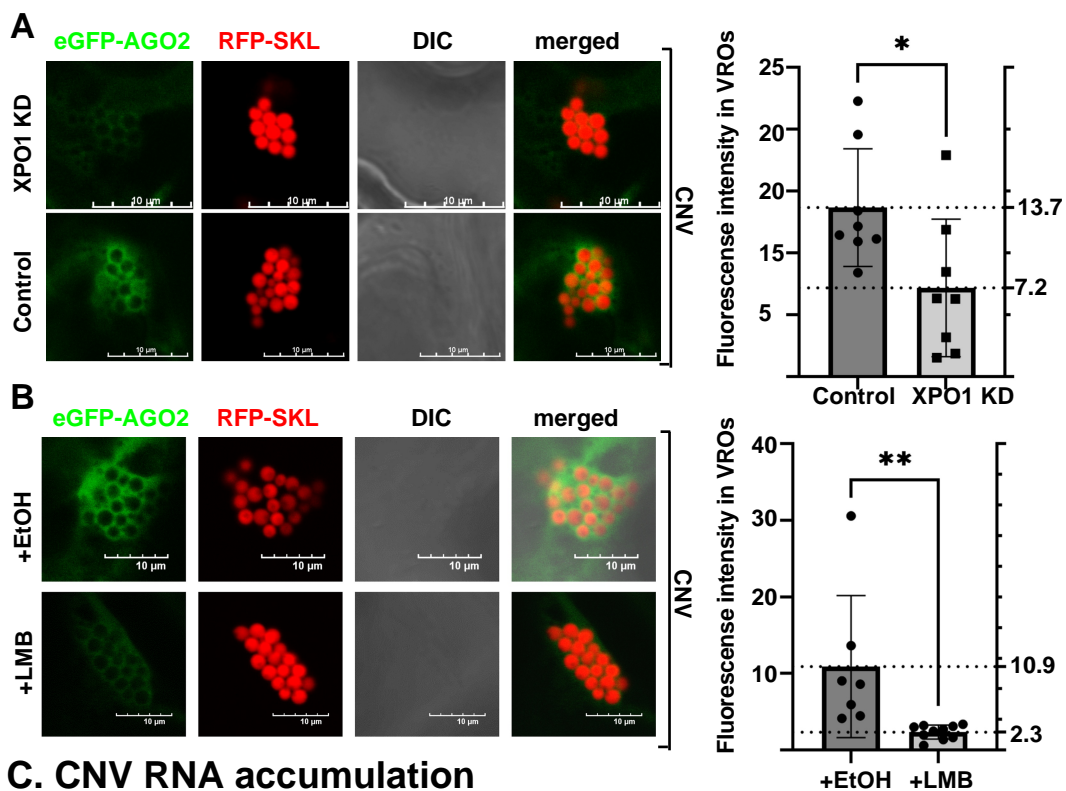

## C. CNV RNA accumulation

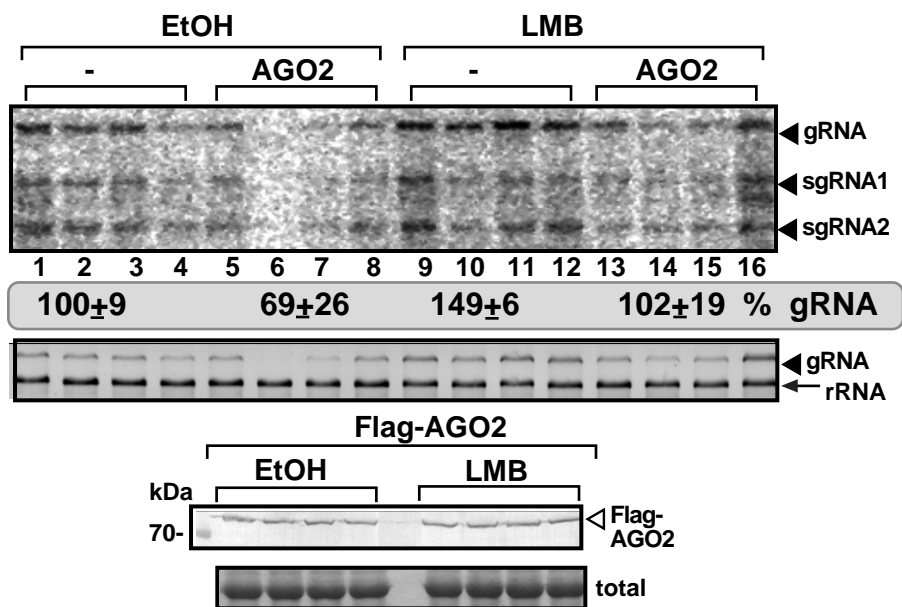

## D. CNV

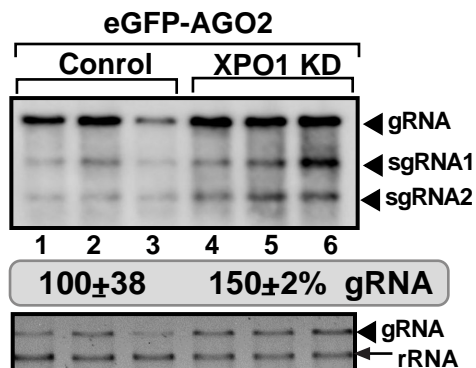

Supplement: S13 Fig — (A) Recruitment of eGFP-AGO2 to VROs is reduced in XPO1 knockdown (KD) N. benthamiana infected with CNV. Left top panel: Confocal microscopic images show poor recruitment of eGFP-AGO2 into VROs (indicated by RFP-SKL) in XPO1 KD N. benthamiana. Bottom panel: Control experiment with N. benthamiana plants agroinfiltrated with the TRV vectors carrying partial GST sequence. Scale bars represent 10 μm. Right panel: Quantification of fluorescent intensity of eGFP-AGO2 in VRO regions was done with Olympus FV3000 FLUO-view software. T-test is used for data analysis utilizing GraphPad Prism 9 (* represents P < 0.1). Error bars represent standard deviation (SD). (B) Recruitment of eGFP-AGO2 to VROs is reduced in N. benthamiana infected with CNV and treated with 400 nM LMB versus 0.5% EtOH. See further details in panel A. (** represents P < 0.01). (C) Transient expression of Flag-AGO2 in N. benthamiana does not inhibit CNV accumulation when leaves are treated with LMB. Note that CNV p20 was not expressed in plants. Top panel: CNV RNA accumulation at 2 dpi was measured by northern blot analysis. N. benthamiana leaves were agroinfiltrated to express Flag-AGO2 (pGD vector as control) and the agroinfiltrated leaves were either infiltrated twice with either 0.5% ethanol (EtOH) as a control or 400 nM of Leptomycin B (LMB). Middle panel: The 18S ribosomal RNA is showed in an agarose gel stained with ethidium-bromide as the loading control. Bottom panel: The expression of Flag-AGO2 was measured by western blot from the above samples using anti-flag antibody. Coomassie Brilliant Blue staining was used for the normalization of total proteins as loading control. (D) Top panel: Accumulation of CNV gRNA and sgRNAs at 2 dpi was measured by northern blot analysis in XPO1-silenced plants expressing eGFP-AGO2. Agroinfiltration to express eGFP-AGO2 (in the absence of p19 silencing suppressor) was performed 7 days after silencing of XPO1 in N. benthamiana, followed two days later by inoculation w [file ppat.1012841.s013.pdf]

S14 FIG

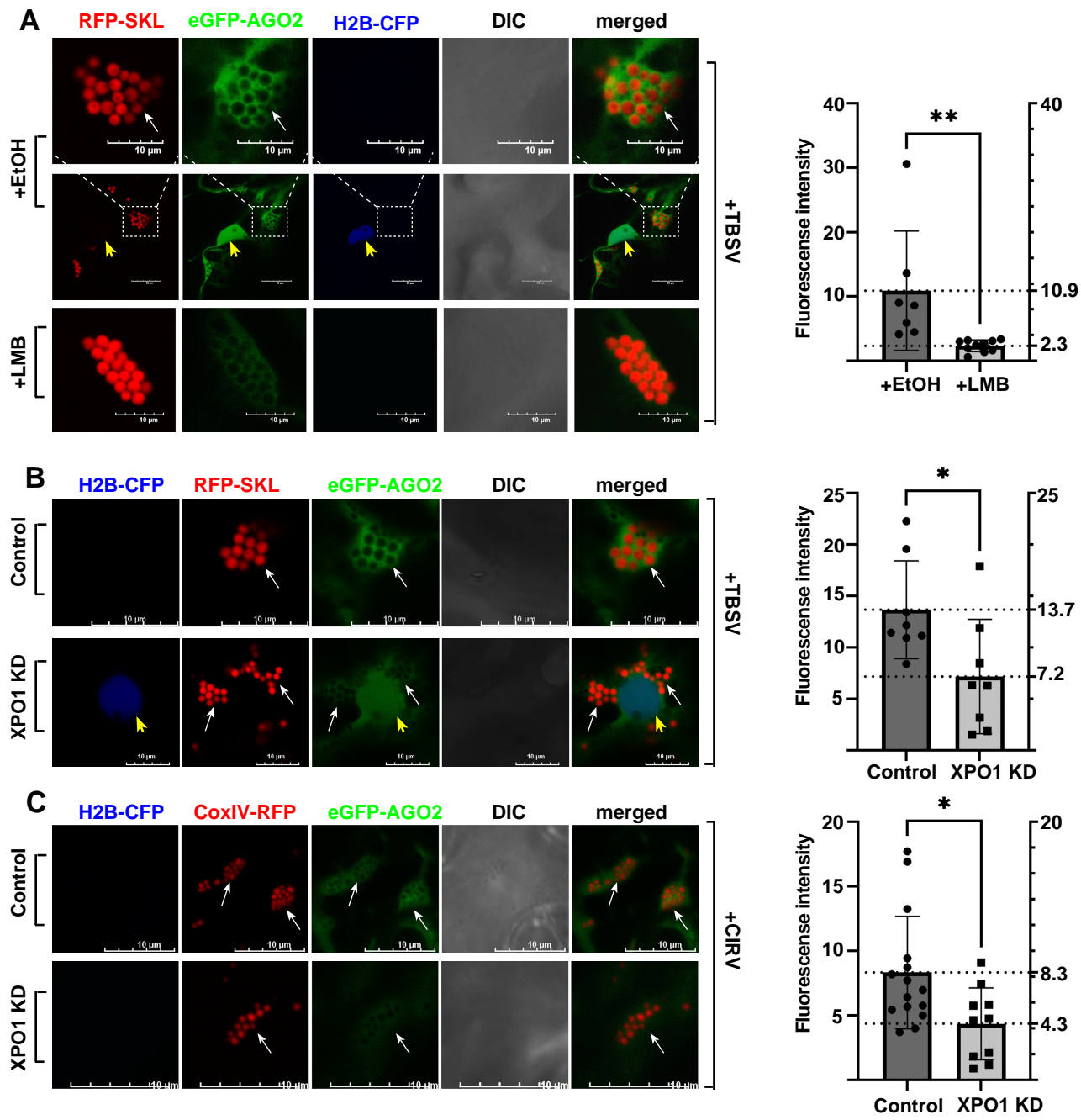

Supplement: S14 Fig — (A-B) Recruitment of eGFP-AGO2 to VROs is reduced in LMB-treated (A) or XPO1 knockdown (KD) (panel B) N. benthamiana infected with TBSV. Left top panel: Confocal microscopic images show poor recruitment of eGFP-AGO2 into VROs (indicated by RFP-SKL) in XPO1 KD H2B-CFP transgenic N. benthamiana. Yellow arrows indicate the nucleus, while VROs are marked with white arrows. Scale bars represent 10 μm. Right panels: Quantification of fluorescent intensity of eGFP-AGO2 in VRO regions was done with Olympus FV3000 FLUO-view software. (C) Recruitment of eGFP-AGO2 to VROs is reduced in XPO1 KD H2B-CFP transgenic N. benthamiana infected with CIRV. CoxIV-RFP marks the clustered mitochondria representing CIRV VROs. See further details in panel A. (** represents P < 0.01). (PDF) [file ppat.1012841.s014.pdf]

S15 FIG

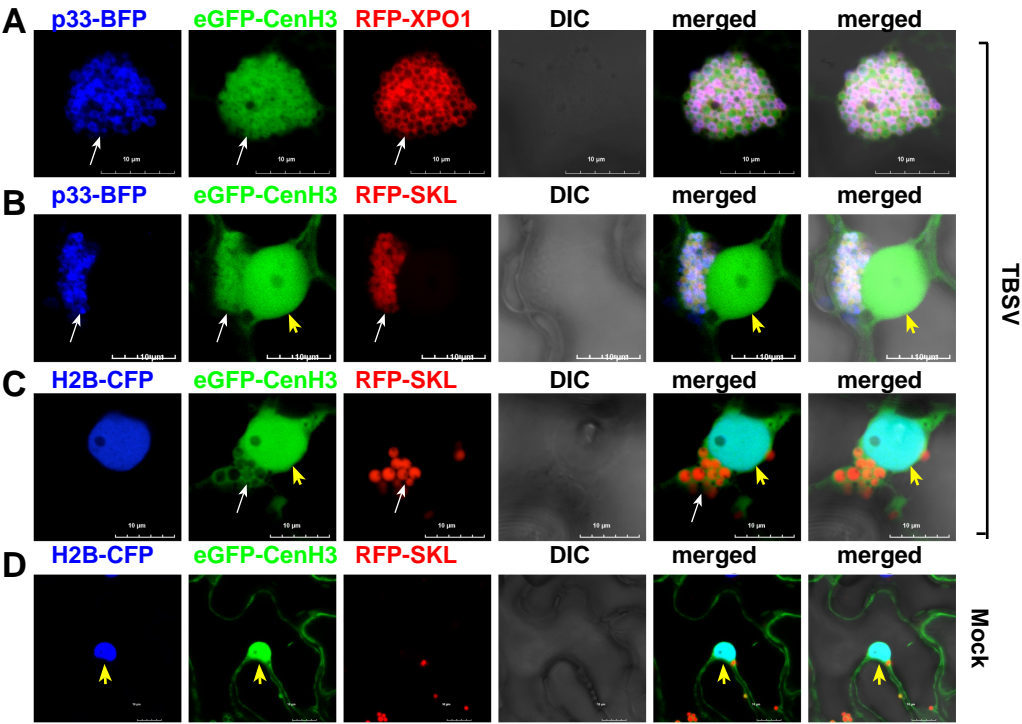

Supplement: S15 Fig — (A-D) Confocal microscopy images show localization of eGFP-CenH3 in VROs in H2B-CFP transgenic N. benthamiana infected with TBSV or mock-treated. RFP-SKL marks the clustered peroxisomes representing TBSV VROs. Yellow arrows indicate the nucleus, while VROs are marked with white arrows. Scale bars represent 10 μm. See further details in S6 Fig. (PDF) [file ppat.1012841.s015.pdf]

S16 FIG

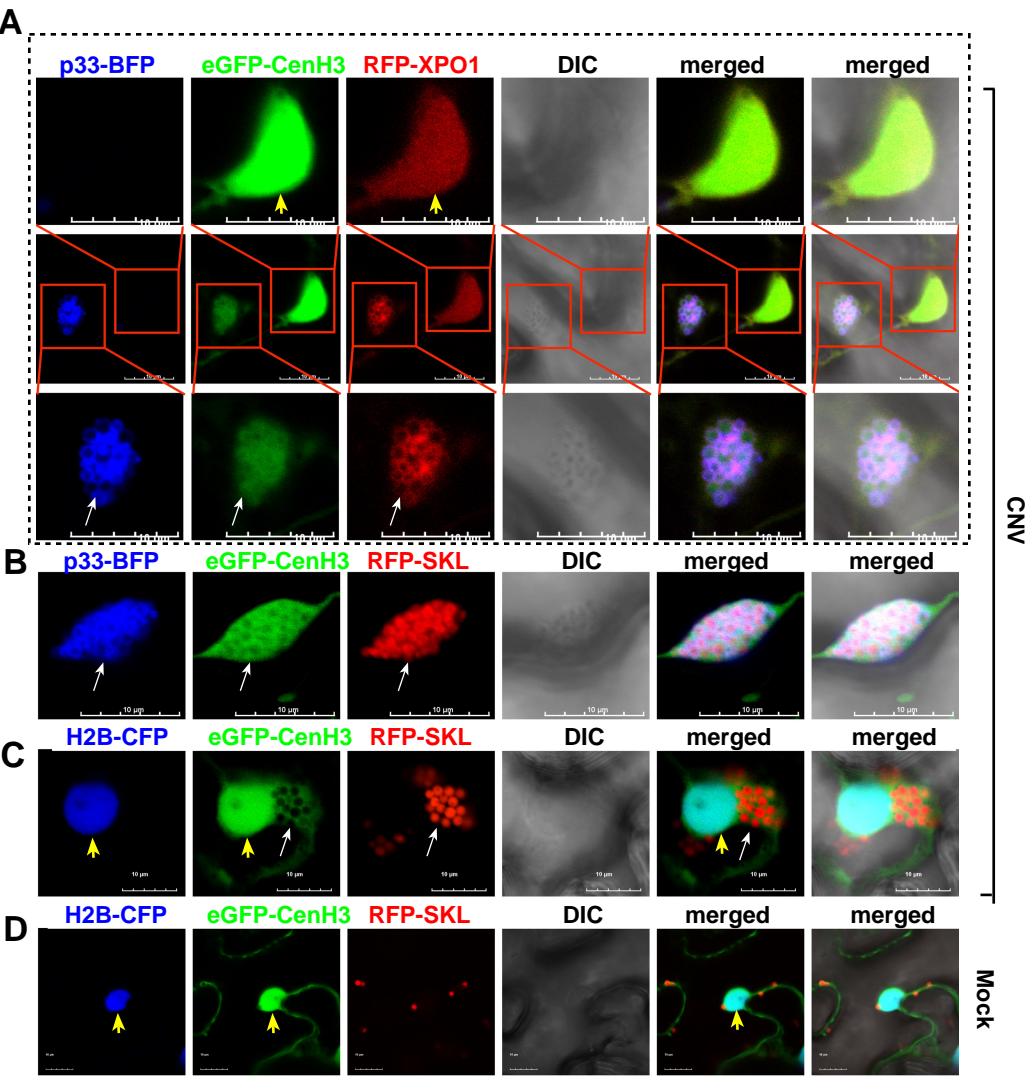

Supplement: S16 Fig — (A-D) Confocal microscopy images show localization of eGFP-CenH3 in VROs in H2B-CFP transgenic N. benthamiana infected with CNV or mock-treated. RFP-SKL marks the clustered peroxisomes representing CNV VROs. Yellow arrows indicate the nucleus, while VROs are marked with white arrows. Scale bars represent 10 μm. See further details in S7 Fig. (PDF) [file ppat.1012841.s016.pdf]

S17 FIG

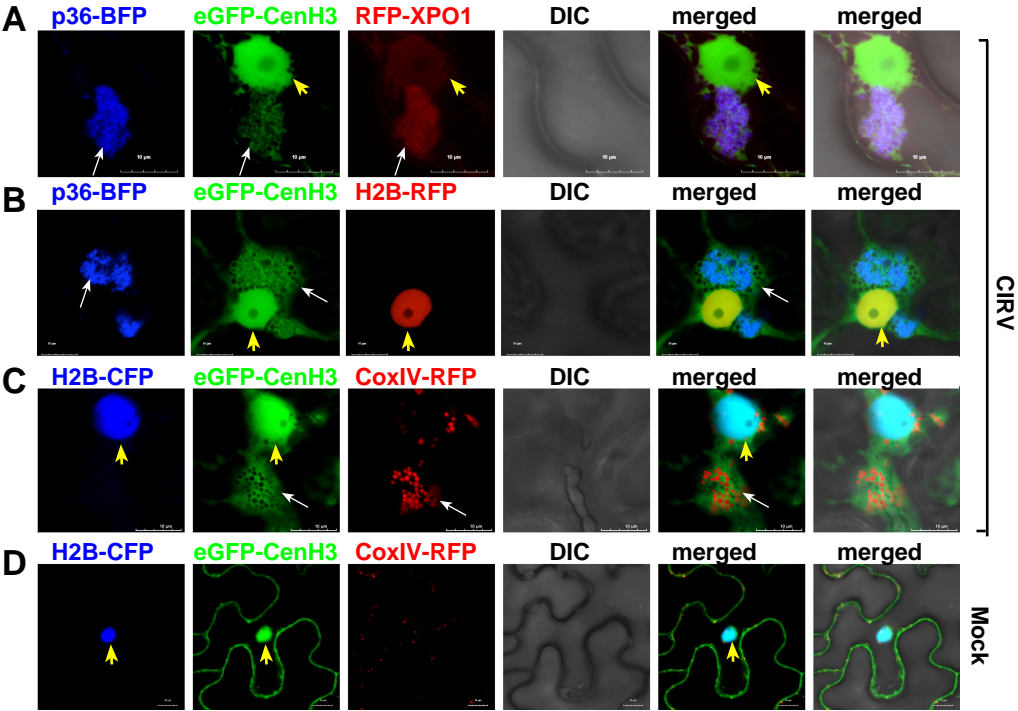

Supplement: S17 Fig — (A-D) Confocal microscopy images show localization of eGFP-CenH3 in VROs in H2B-CFP transgenic N. benthamiana infected with CIRV. CoxIV-RFP marks the clustered mitochondria representing CIRV VROs. Yellow arrows indicate the nucleus, while VROs are marked with white arrows. Scale bars represent 10 μm. See further details in S8 Fig. (PDF) [file ppat.1012841.s017.pdf]

**S18 FIG**

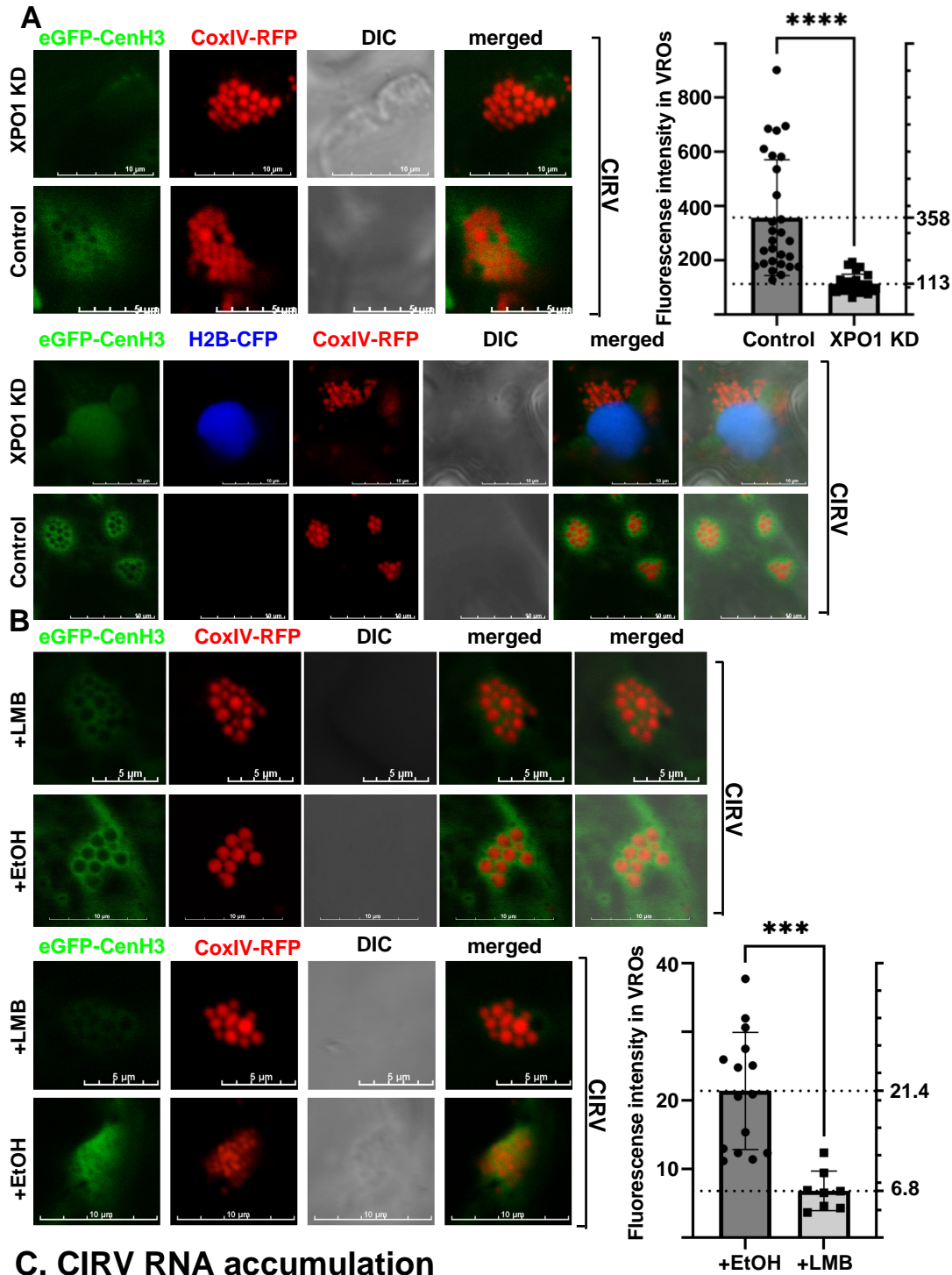

### C. CIRV RNA accumulation

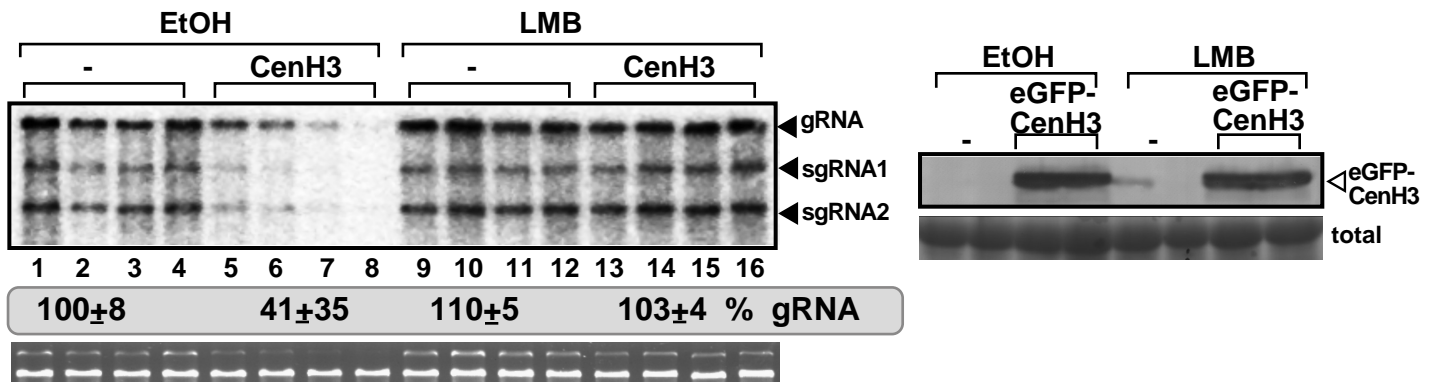

Supplement: S18 Fig — (A) Recruitment of eGFP-CenH3 to CIRV VROs is reduced in XPO1 knockdown (KD) N. benthamiana infected with CIRV. Left top panel: Confocal microscopic images show poor recruitment of eGFP-CenH3 into VROs (indicated by CoxIV-RFP) in XPO1 KD N. benthamiana. Bottom panel: Control experiment with N. benthamiana plants agroinfiltrated with the TRV vectors carrying partial GST sequence. Scale bars represent 10 μm. Right panel: Quantification of fluorescent intensity of eGFP-CenH3 in VRO regions was done with Olympus FV3000 FLUO-view software. T-test is used for data analysis utilizing GraphPad Prism 9 (**** represents P < 0.0001). Error bars represent standard deviation (SD). (B) Recruitment of eGFP-CenH3 to VROs is reduced in N. benthamiana infected with CIRV and treated with 400 nM LMB versus 0.5% EtOH. See further details in panel A. (*** represents P < 0.001). (C) Transient expression of Flag- CenH3 in N. benthamiana does not inhibit CIRV accumulation when leaves were treated with LMB versus EtOH. Top panel: CIRV RNA accumulation at 2.5 dpi was measured by northern blot analysis. N. benthamiana leaves were agroinfiltrated to express Flag-CenH3 (pGD vector as control) and the agroinfiltrated leaves were either infiltrated with 0.5% ethanol (EtOH) as a control or 400 nM of Leptomycin B (LMB). Middle panel: The 18S ribosomal RNA is showed in an agarose gel stained with ethidium-bromide as the loading control. Bottom right panel: The expression of Flag-CenH3 was measured by western blot from the above samples using anti-flag antibody. Coomassie Brilliant Blue staining was used for the normalization of total proteins as loading control. Each experiment was repeated three times. (PDF) [file ppat.1012841.s018.pdf]

# S19 FIG

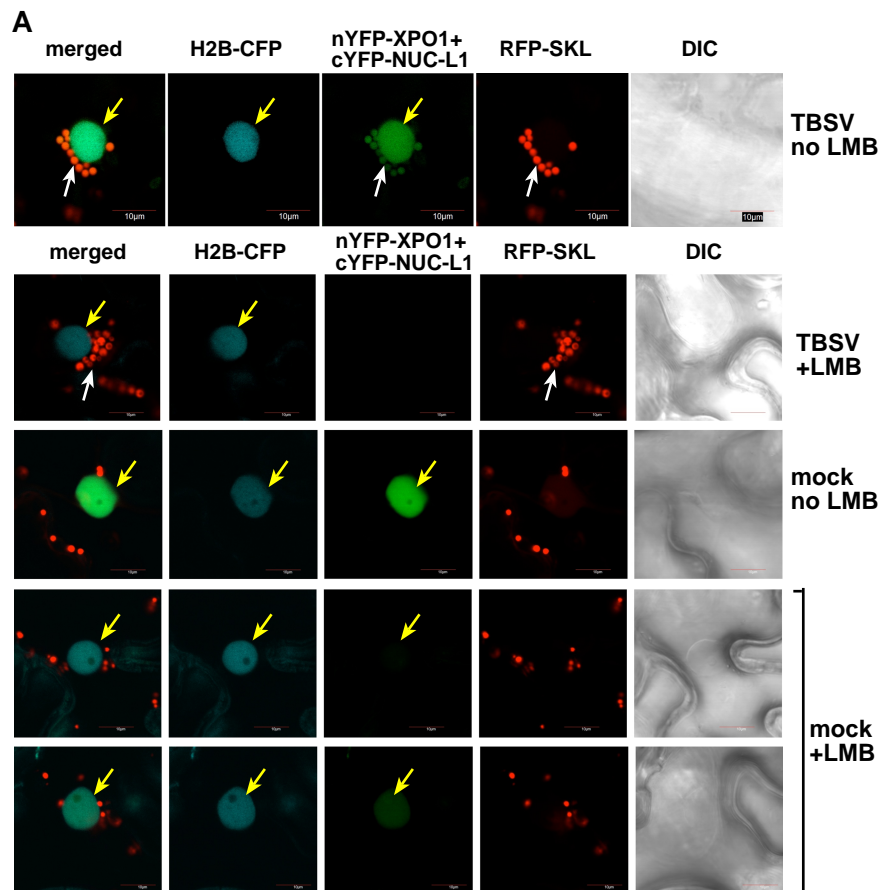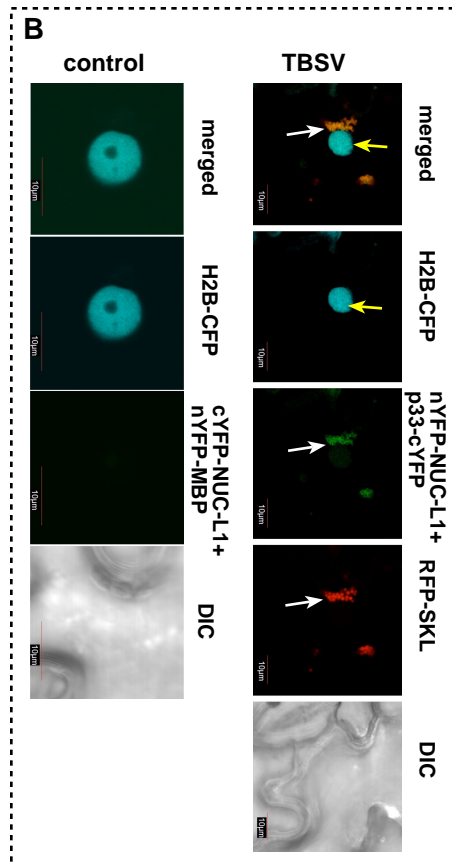

## C. CNV

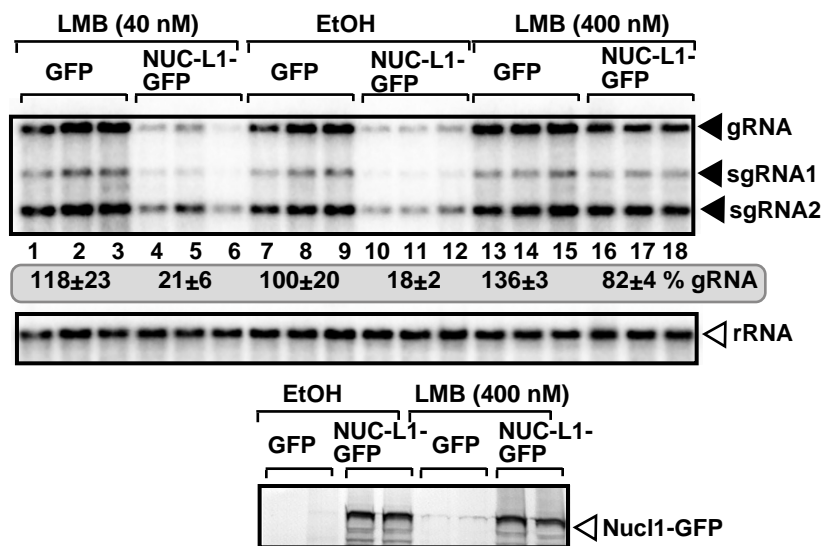

Supplement: S19 Fig — (A) Interaction between XPO1 and NUC-L1 (NUCLEOLIN) and recruitment of NUC-L1 to TBSV VROs is dampened by treatment of leaves with LMB versus EtOH. Top panel: Confocal microscopic images show interaction between XPO1 and NUC-L1 and recruitment of NUC-L1 to TBSV VROs based on BiFC assay. TBSV VROs are indicated by RFP-SKL in H2B-CFP transgenic N. benthamiana. Yellow arrows indicate the nucleus marked with H2B-CFP, while VROs are marked with white arrows. Second panel: Similar BiFC assay as in panel A, except leaves were treated with LMB or EtOH. Control experiments were done with H2B-CFP transgenic N. benthamiana plants treated with LMB or not treated. Scale bars represent 10 μm. (B) BiFC assay shows interaction between nYFP-NUC-L1 and p33-cYFP in VROs in H2B-CFP transgenic N. benthamiana infected with TBSV. (C) Transient expression of NUC-L1-GFP in N. benthamiana does not inhibit CNV accumulation when leaves were treated with LMB versus EtOH. Top panel: CNV RNA accumulation at 2 dpi was measured by northern blot analysis. N. benthamiana leaves were agroinfiltrated to express NUC-L1-GFP (GFP as control) and the agroinfiltrated leaves were either infiltrated with 0.5% ethanol (EtOH) as a control or 40 or 400 nM LMB. Middle panel: The 18S ribosomal RNA is showed in northern blot as the loading control. Bottom right panel: The expression of NUC-L1-GFP was measured by western blot from the above samples using anti-GFP antibody. Each experiment was repeated three times. (PDF) [file ppat.1012841.s019.pdf]

S20 FIG

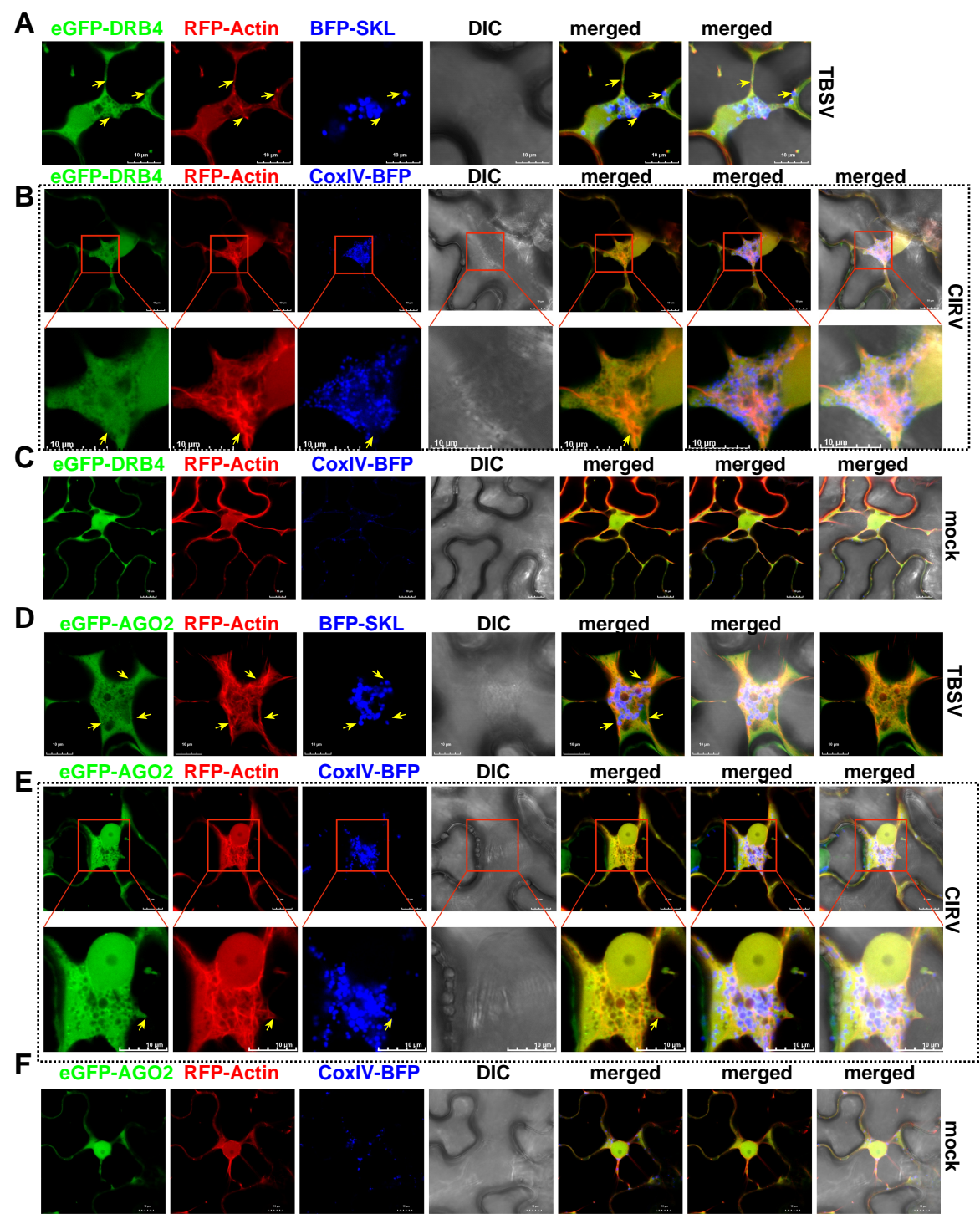

Supplement: S20 Fig — (A-B) Confocal microscopy images show co-localization of eGFP-DRB4 with RFP-Actin filaments and VROs, indicated by BFP-SKL during TBSV replication or by CoxIV-BFP mitochondrial marker during CIRV replication in N. benthamiana. The actin filaments were visualized by agro-expression of Lifeact, which binds to filamentous actin (F-actin) in plant cells. The actin filaments co-localized with eGFP-DRB4 are indicated by yellow arrows. The scale bar represents 10 μm. The enlarged images from the red boxed areas contain CIRV VROs and actin filaments in the vicinity of the nucleus. (C) Localization of eGFP-DRB4, RFP-Actin, and CoxIV-BFP mitochondrial marker in mock-treated N. benthamiana. The scale bar represents 10 μm. (D-E) Confocal microscopy images show the co-localization of eGFP-AGO2 with RFP-Actin and TBSV or CIRV VROs in N. benthamiana cells. TBSV VROs are marked with SKL-BFP peroxisomal marker, whereas CIRV VROs are marked with CoxIV-BFP mitochondrial marker. The scale bar represents 10 μm. (F) Localization of eGFP-AGO2, RFP-Actin, and CoxIV-BFP mitochondrial marker in mock-treated N. benthamiana. The scale bar represents 10 μm. Each experiment was repeated three times. (PDF) [file ppat.1012841.s020.pdf]

S21 FIG

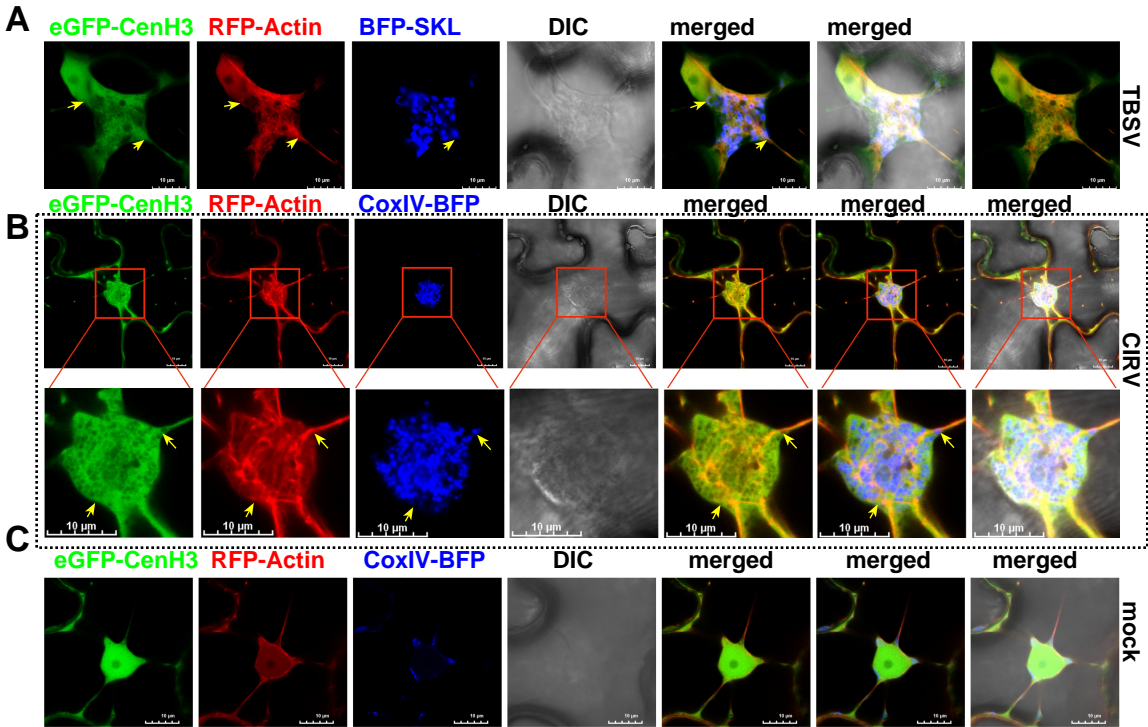

Supplement: S21 Fig — (A-B) Confocal microscopy images show co-localization of eGFP-CenH3 with RFP-Actin filaments and VROs, indicated by BFP-SKL during TBSV replication or by CoxIV-BFP mitochondrial marker during CIRV replication in N. benthamiana. The actin filaments were visualized by agro-expression of Lifeact, which binds to filamentous actin (F-actin) in plant cells. The actin filaments co-localized with eGFP-CenH3 are indicated by yellow arrows. The scale bar represents 10 μm. The enlarged images from the red boxed areas contain CIRV VROs and actin filaments. (C) Localization of eGFP-CenH3, RFP-Actin, and CoxIV-BFP mitochondrial marker in mock-treated N. benthamiana. The scale bar represents 10 μm. (PDF) [file ppat.1012841.s021.pdf]

S22 FIG

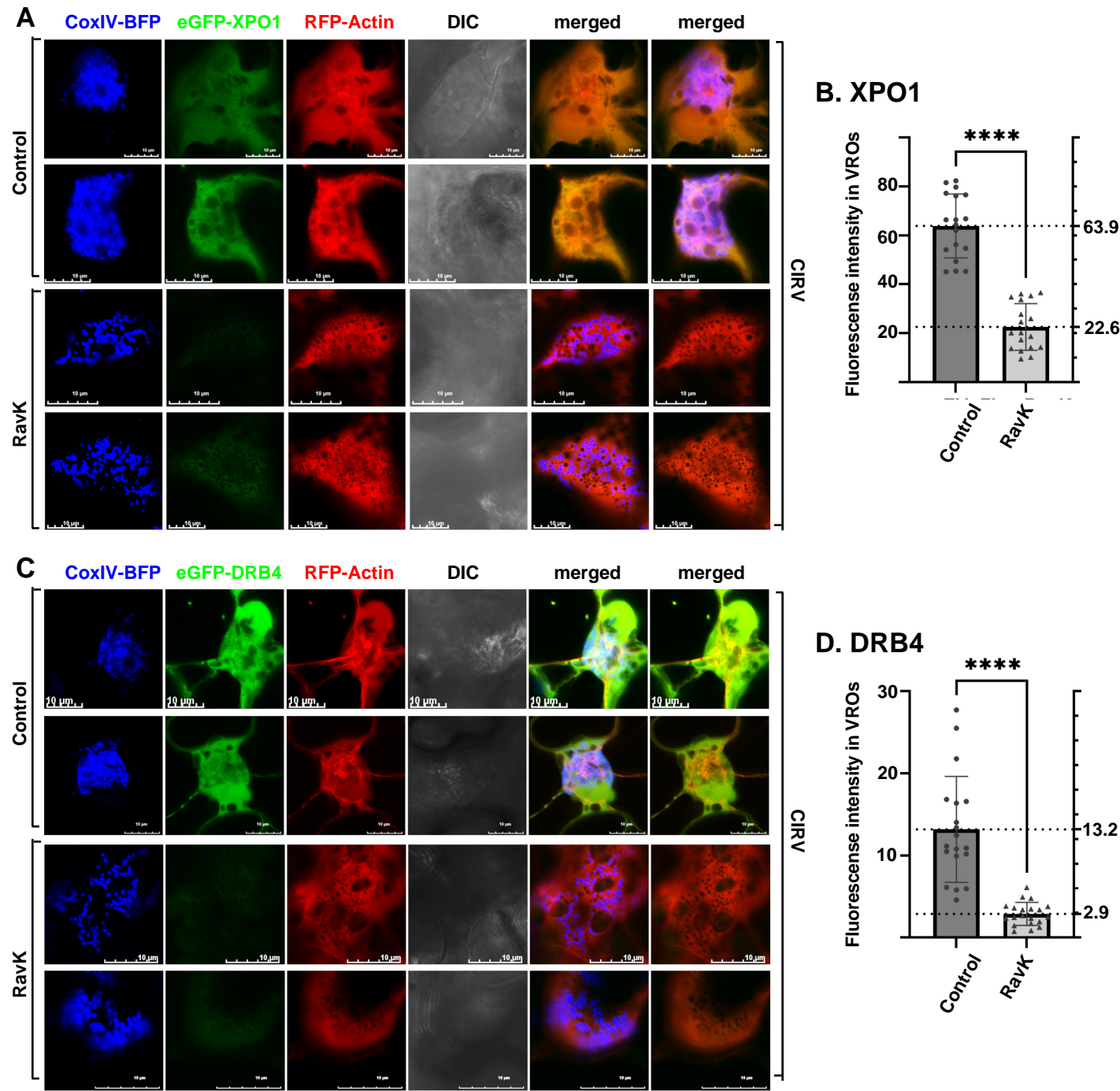

Supplement: S22 Fig — Confocal microscopy images show the poor recruitment of eGFP-XPO1 (A), or eGFP-DRB4 (C) into CIRV VROs when the actin filaments were destroyed by the transient expression of RavK effector of Legionella bacterium from a plasmid via agroinfiltration in N. benthamiana. Top two panels in (A and C) show the control confocal images without the expression of RavK effector in N. benthamiana. Scale bars represent 10 μm. Quantification of VRO-localized eGFP-XPO1 (B) or eGFP-DRB4 (D) is shown in graphs. The fluorescent intensity in VROs is quantified by Olympus FV3000 FLUO-view software. T-test is used for data analysis utilizing GraphPad Prism 9 (**** represents P < 0.0001). Error bars represent standard deviation (SD). Each experiment was repeated three times. (PDF) [file ppat.1012841.s022.pdf]

S23 FIG

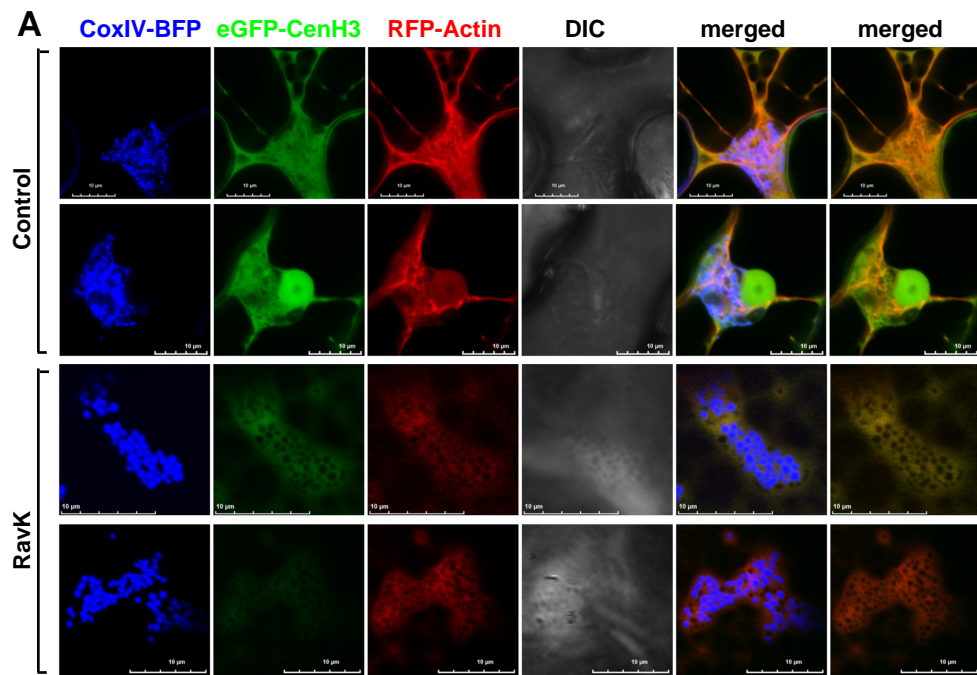

**B. CenH3**

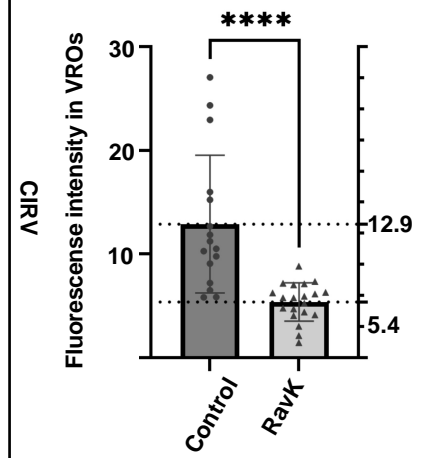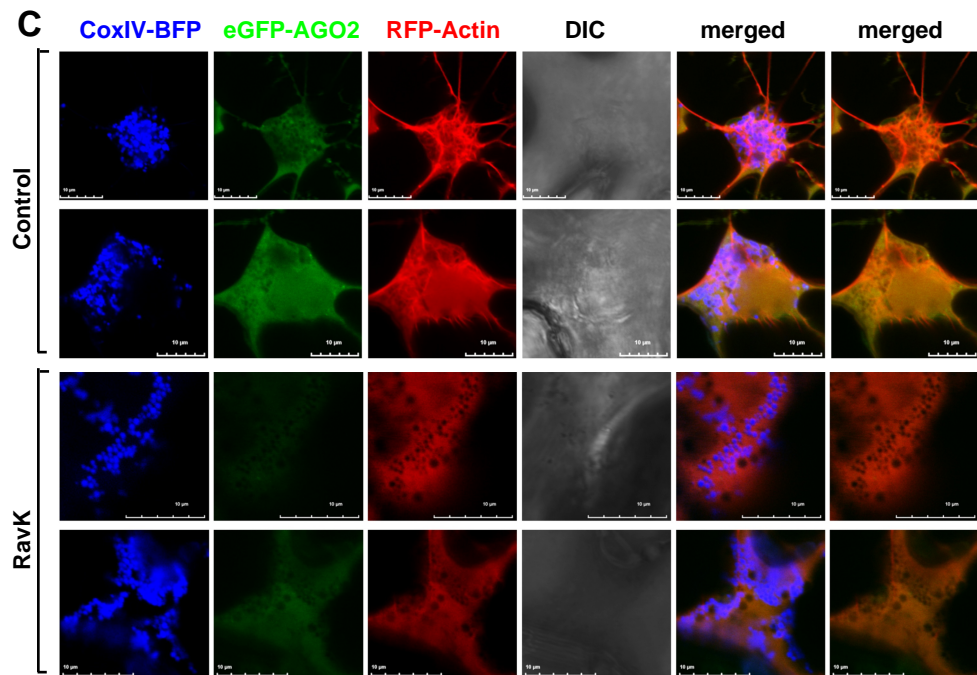

**D. AGO2**

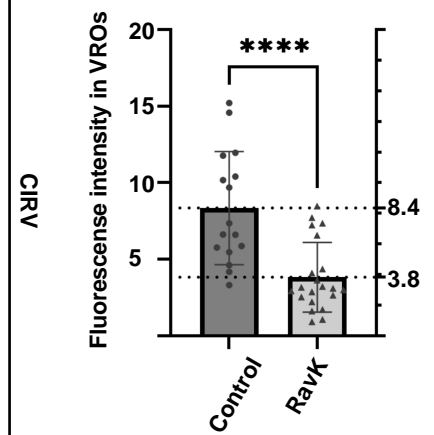

Supplement: S23 Fig — Confocal microscopy images show the poor recruitment of eGFP-CenH3 (A), or eGFP-AGO2 (C) into CIRV VROs when the actin filaments were destroyed by the transient expression of RavK effector of Legionella bacterium from a plasmid via agroinfiltration in N. benthamiana. See further details in S16 Fig. Scale bars represent 10 μm. Quantification of VRO-localized eGFP-CenH3 (B) or eGFP-AGO2 (D) is shown in graphs. The fluorescent intensity in VROs is quantified by Olympus FV3000 FLUO-view software. T-test is used for data analysis utilizing GraphPad Prism 9 (**** represents P < 0.0001). Error bars represent standard deviation (SD). Each experiment was repeated three times. (PDF) [file ppat.1012841.s023.pdf]

S24 FIG

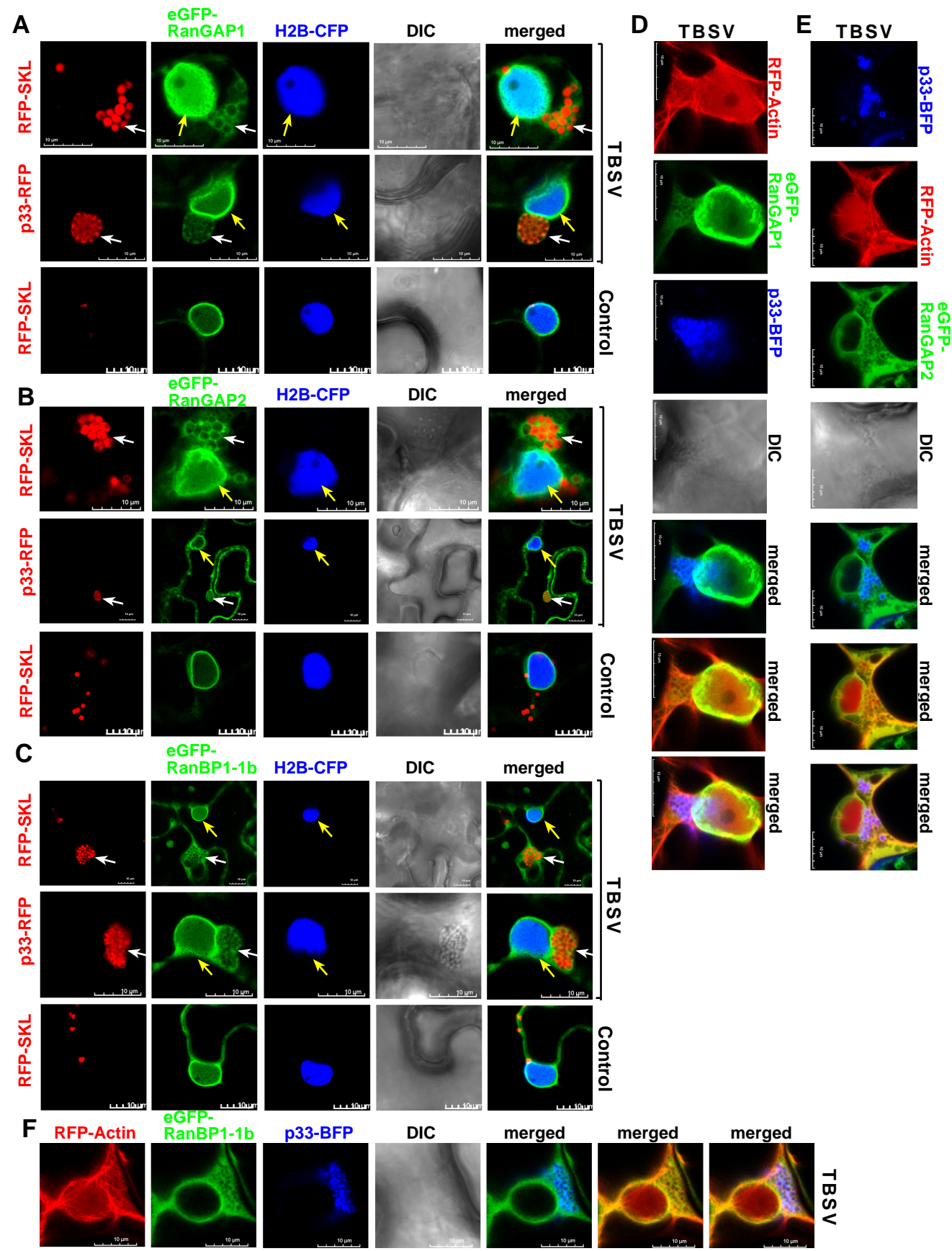

Supplement: S24 Fig — Confocal microscopy images show the recruitment of eGFP-RanGAP1 (A), eGFP-RanGAP2 (B), or eGFP-RanBP1-1b (C) into VROs during TBSV replication. The bottom images in panels A, B and C show the subcellular localizations of eGFP-RanGAP1, eGFP-RanGAP2, or eGFP-RanBP1-1b in mock inoculated control plant cells. Yellow arrows indicate the nucleus, while VROs are marked with white arrows. Note these images were taken using H2B-CFP transgenic N. benthamiana marking the nucleus. Confocal microscopy images also show the associations of eGFP-RanGAP1 (D), eGFP-RanGAP2 (E), eGFP-RanBP1-1b (F) and p33-BFP replication protein with actin filament networks during TBSV replication. Scale bars represent 10 μm. (PDF) [file ppat.1012841.s024.pdf]

S25 FIG

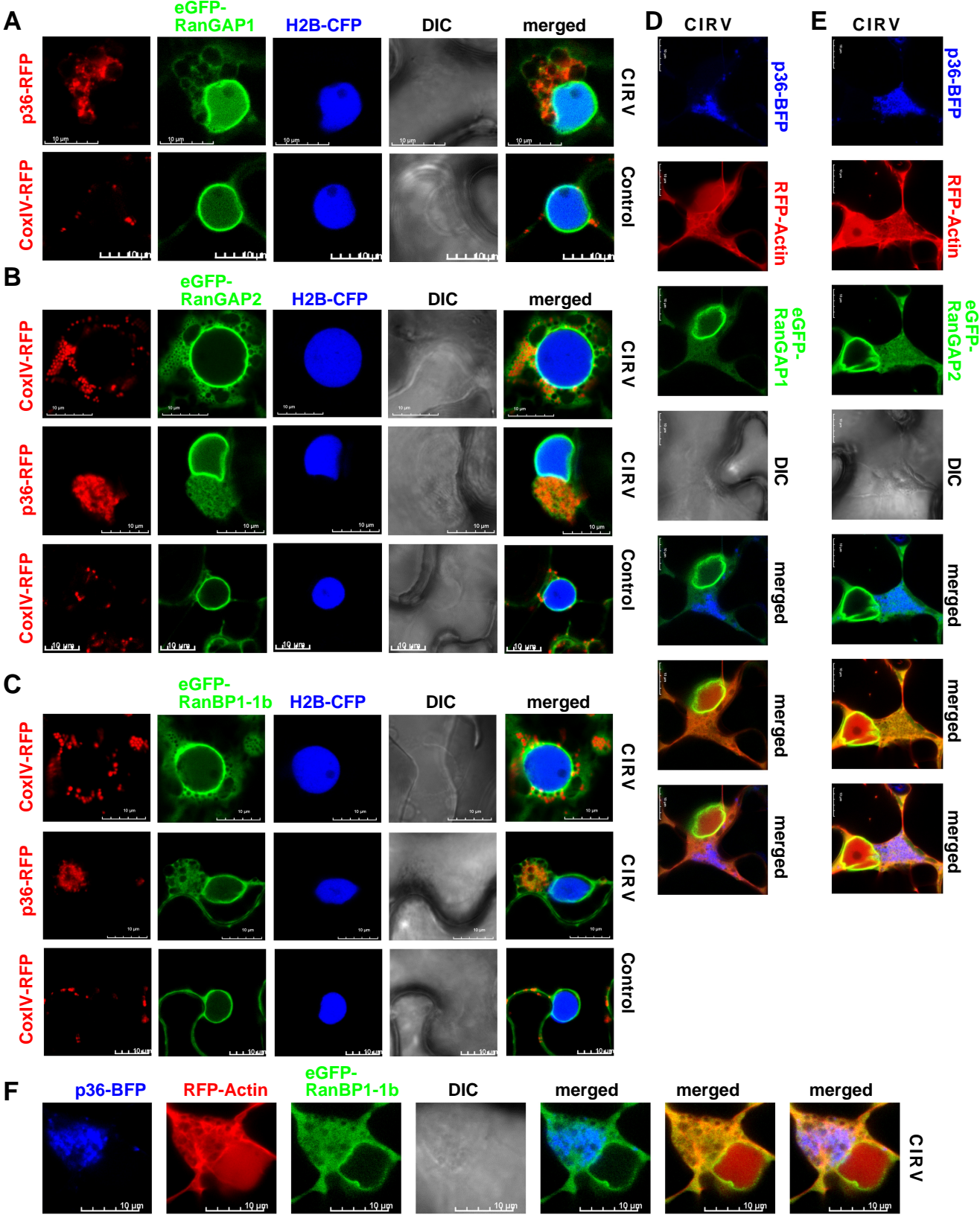

Supplement: S25 Fig — Confocal microscopy images show the recruitment of eGFP-RanGAP1 (A), eGFP-RanGAP2 (B), or eGFP-RanBP1-1b (C) into VROs during CIRV replication. The bottom images in panel A, B and C show the subcellular localizations of eGFP-RanGAP1, eGFP-RanGAP2, or eGFP-RanBP1-1b in control plant cells. The CIRV VROs are labeled with either CoxIV-BFP mitochondrial marker or p36-BFP replication protein during CIRV replication. Note these images were taken using H2B-CFP transgenic N. benthamiana marking the nucleus. Confocal microscopy images also show the associations of eGFP-RanGAP1 (D), eGFP-RanGAP2 (E), eGFP-RanBP1-1b (F) and CIRV p36-BFP replication protein with actin filament networks during CIRV replication. Scale bars represent 10 μm. Each experiment was repeated three times. (PDF) [file ppat.1012841.s025.pdf]

**S26 FIG**

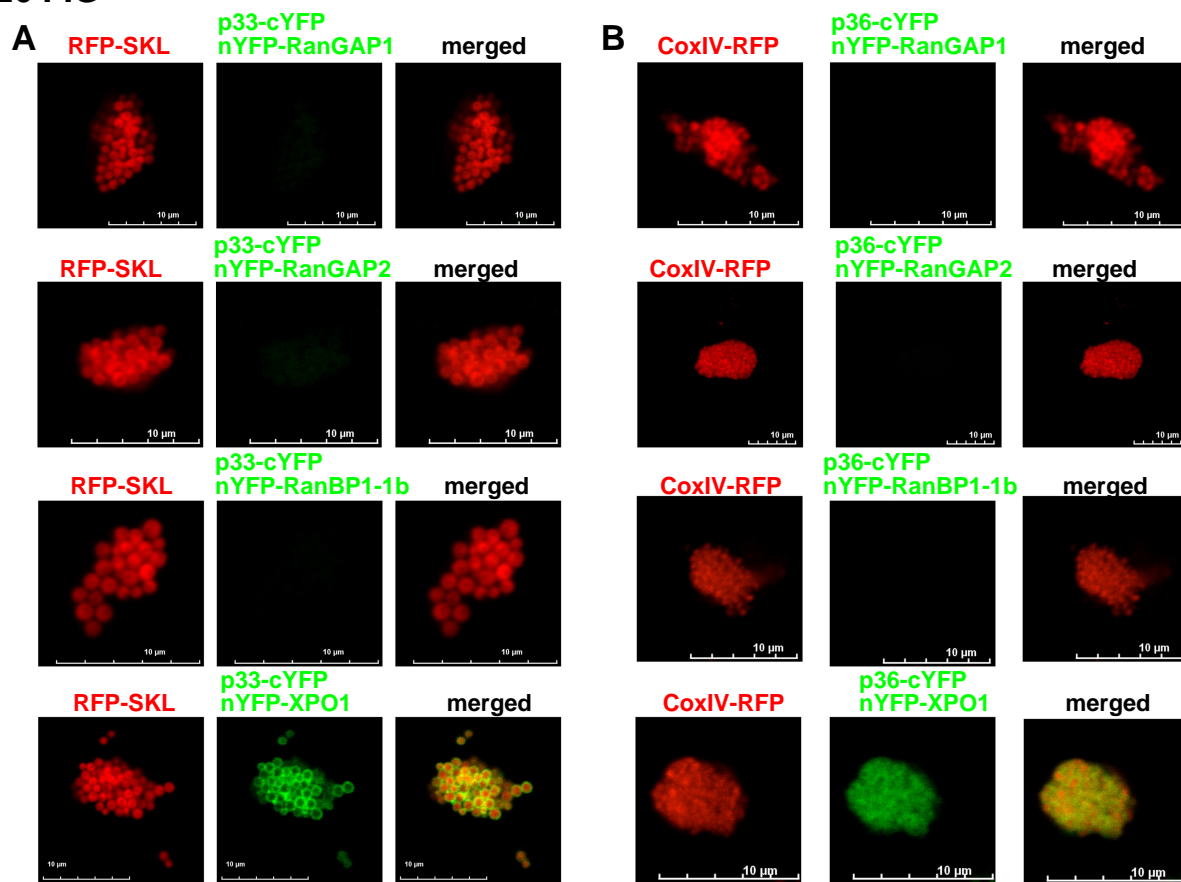

Supplement: S26 Fig — (A) TBSV p33 replication protein does not interact with regulatory co-factors of XPO1. From the first to the third panels: BiFC-based interaction results of nYFP-tagged RanGAP1, RanGAP2, and RanBP1-1b with p33-cYFP are shown during TBSV replication. Bottom panel: BiFC assay shows that p33-cYFP interacts with nYFP-XPO1 during TBSV replication as the positive control. (B) CIRV p36 replication protein does not interact with regulatory co-factors of XPO1. From the first to the third panels: BiFC-based interaction results of nYFP-tagged RanGAP1, RanGAP2, and RanBP1-1b with p36-cYFP are shown during CIRV replication. Bottom panel: BiFC assay shows that p36-cYFP interacts with nYFP-XPO1 during CIRV replication as the positive control. Scale bars represent 10 μm. (PDF) [file ppat.1012841.s026.pdf]

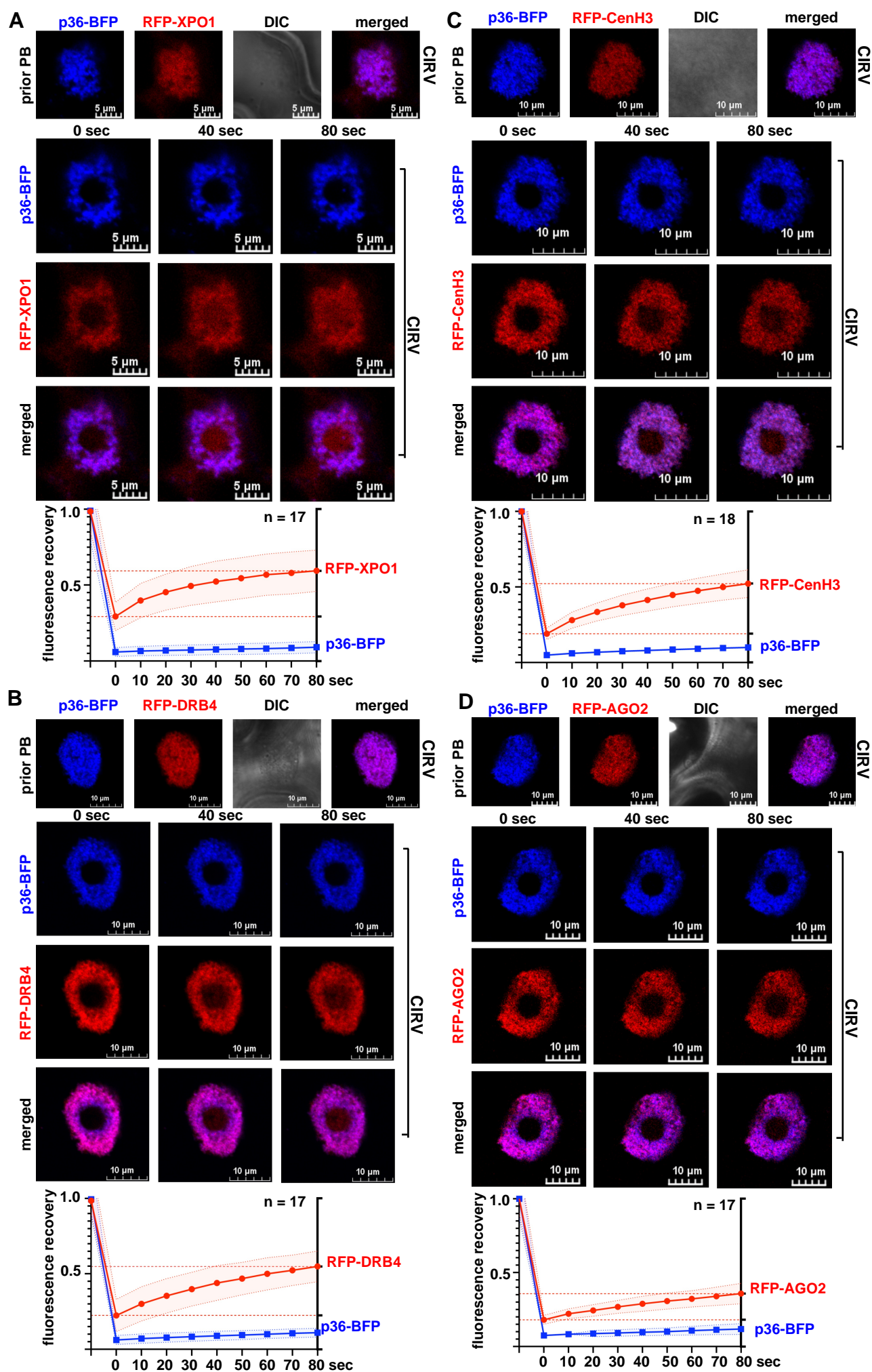

Supplement: S27 Fig — (A) FRAP analysis shows partial fluorescence recovery of RFP-XPO1 after photobleaching in a single CIRV VRO in N. benthamiana during CIRV replication. Top panel: Co-localization of CIRV p36-BFP replication protein and RFP-XPO1 in VRO before photobleaching. Middle panels: Confocal microscopy images show partial fluorescence recovery of RFP-XPO1 at 0-, 40-, and 80-seconds post photobleaching during CIRV replication. Note that signals of CIRV p36-BFP did not recover in the FRAP assay because it is a membrane-bound protein. Bottom panel: The graph shows time course analysis of FRAP data on RFP-XPO1 signal recovery in individual CIRV VROs. Sample size n is annotated in the figure. Shaded area represents SD. Scale bars represent 5 μm. Similar FRAP analysis of RFP-DRB4 (panel B), RFP-CenH3 (panel C), and RFP-AGO2 (panel D) are also shown. See further details in panel A. Each experiment was repeated three times. (PDF) [file ppat.1012841.s027.pdf]
