# Supplementary material for: Mobilization of nuclear antiviral factors by exportin XPO1 via the actin network inhibits RNA virus replication
Source: PLoS Pathog. 2025 Aug 19;21(8):e1012841. doi: 10.1371/journal.ppat.1012841 (PMC12393752; doi:10.1371/journal.ppat.1012841)
Supplement: S28 Fig — (A) Confocal images show the co-partitioning of the purified mRFP-DRB4 and eGFP-p33C in droplets in the presence of 2.5% PEG8000. The top and bottom panels show droplets formed by different amounts of mRFP-DRB4 as indicated. Scale bars represent 10 µm. (B) Note that purified mRFP-DRB4 and eGFP-p33C form droplets under the same conditions as in panel A. (C) Confocal images show the co-partitioning of the purified mRFP-CenH3 and eGFP-p33C in droplets in the presence of 2.5% PEG8000. The top and bottom panels show droplets formed by different amounts of mRFP-CenH3 as indicated. Scale bars represent 10 µm. (D) Note that purified mRFP-CenH3 forms a few droplets under the same conditions as in panel C. Each experiment was repeated three times. (PDF) [file ppat.1012841.s028.pdf]

S28 FIG

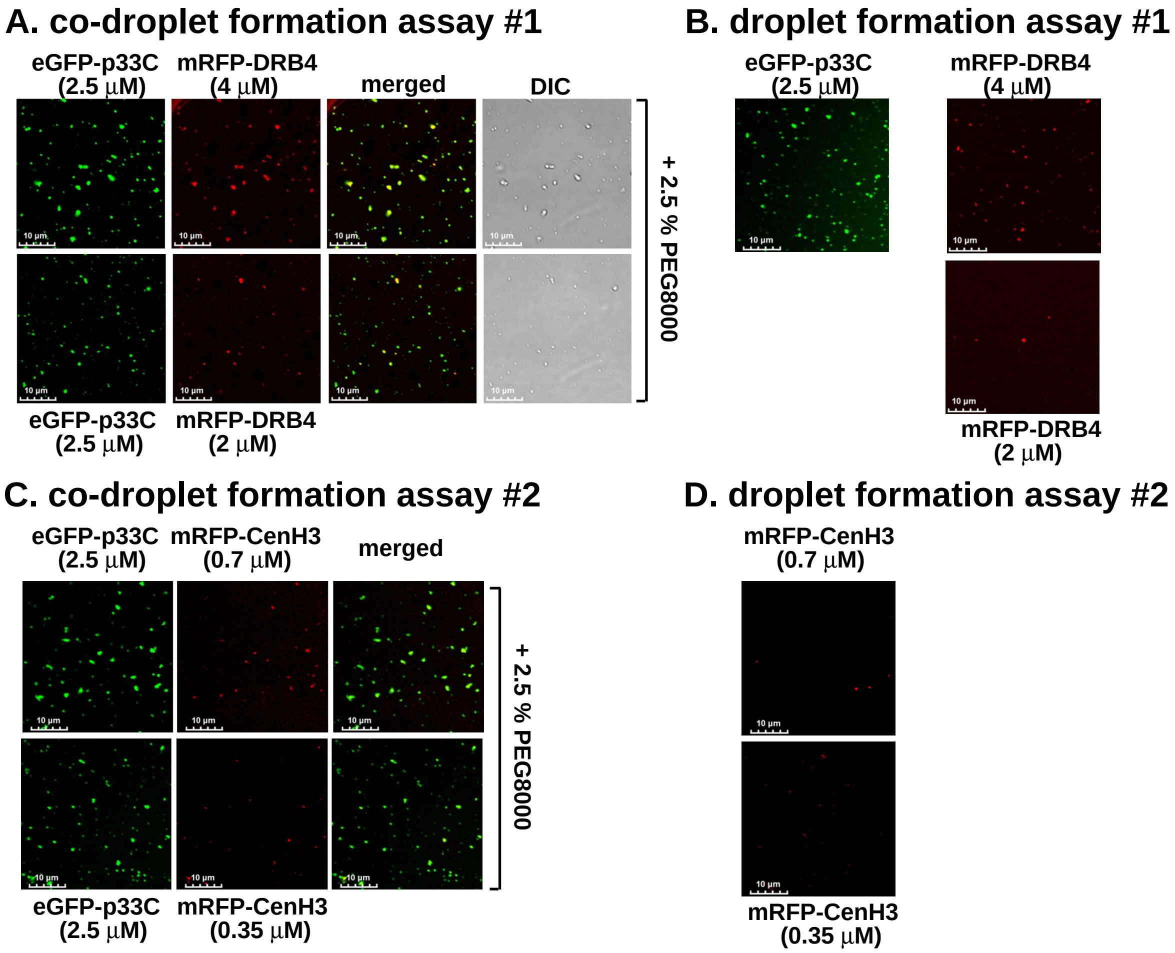
A. co-droplet formation assay #1
eGFP-p33C (2.5 μM)
mRFP-DRB4 (4 μM)
merged
DIC
10 μm
+ 2.5 % PEG8000
eGFP-p33C (2.5 μM)
mRFP-DRB4 (2 μM)
B. droplet formation assay #1
eGFP-p33C (2.5 μM)
mRFP-DRB4 (4 μM)
mRFP-DRB4 (2 μM)
C. co-droplet formation assay #2
eGFP-p33C (2.5 μM)
mRFP-CenH3 (0.7 μM)
merged
eGFP-p33C (2.5 μM)
mRFP-CenH3 (0.35 μM)
D. droplet formation assay #2
mRFP-CenH3 (0.7 μM)
mRFP-CenH3 (0.35 μM)
