# Supplementary material for: Mobilization of nuclear antiviral factors by exportin XPO1 via the actin network inhibits RNA virus replication
Source: PLoS Pathog. 2025 Aug 19;21(8):e1012841. doi: 10.1371/journal.ppat.1012841 (PMC12393752; doi:10.1371/journal.ppat.1012841)
Supplement: S1 Text — (DOC) [file ppat.1012841.s029.doc]

f[

**S1_Text**

**Supplementary Materials and Methods**

**Plant materials and yeast strains.** *Nicotiana benthamiana* wt and H2b-CFP or H2b-RFP transgenic plants were potted in soil and grown in greenhouse for two weeks as seedlings and then moved to growth chamber under a 16-hours-light/ 8-hours-dark cycle at 25 ℃. The yeast strain *Saccharomyces cerevisiae* BY4741 (MATa his3Δ1 leu2Δ0 met15Δ0 ura3Δ0) was purchased from Open Biosystem and stored in -80℃. The temperature-sensitive (ts) mutant of Srm1p in *S. cerecisiae* is a generous gift from Charles Boone [104].

The coding sequences of related genes were downloaded from NCBI and TAIR. *NbXPO1* (Accession number: LC434544), *AtXPO1a* (Accession number: AT5G17020), *NbAGO2* (Accession number: XM_009784216.1), *AtDRB4* (Accession number: AT3G62800), *NbRanGAP1* (Accession number: EF396238.1), *NbRanGAP2* (Accession number: EF396237.1), *NbRanBP1-1b* (Accession number: LC421998.1). Note that *AtXPO1a* (accession number: AT5G17020) was used for expression in *N. benthamiana*.

**Purification of recombinant proteins from *E. coli*.** Recombinant proteins GST-AtXpo1, GST, MBP-p33, MBP-p92 were expressed in *E. coli* and affinity-purified as described [29]. Briefly, *E. coli* strain BL21 (DE3) CodonPlus (Stratagene) cells were transformed with expression plasmids to express the recombinant proteins. The transformed *E. coli* cells were cultured at 37°C for 16h, followed by dilution of the culture to OD_600_ 0.2 with fresh media. The *E. coli* culture was then incubated at 37°C until its OD_600_ 1.0. The culture was supplemented with isopropyl-β-D-thiogalactopyranoside (IPTG) and incubated at 16°C for 8 h. The *E. coli* cells were then collected by centrifugation at 5,000 rpm at 4°C for 5 min, followed by the resuspension with ice-cold column buffer [20mM HEPES (pH7.4), 25 mM NaCl, 1 mM EDTA (pH 8.0)] containing 10 mM β-mercaptoethanol and 1 μg of RNase A for each 4 ml of *E. coli* cells suspension. After sonication on ice, the cell lysates were centrifuged at 15,000 rpm at 4°C for 15 min. The obtained supernatant was incubated with either GST bind resin (EMD Millipore) for GST fusion proteins or amylose resin (NEB) for MBP fusion proteins, respectively, at 4°C for 2 h. After the resins were washed with ice-cold column buffer, elution of the recombinant protein was performed with column buffer containing 10 mM glutathione and 1 mM DTT in pH 7.5 for GST fusion proteins or 0.36% [W/V] maltose and 1 mM DTT for MBP fusion proteins.

**Protein co-purification assay.** To test interaction between TBSV p33 and XPO1, a co-purification assay was conducted based on protein expression in yeast (BY4741). Yeast was co-transformed with plasmid combination of UpYES-6xHis-XPO1, LpGAD-CUP-Flag-p92 and HpGBK-CUP-Flag-p33/Gal: DI72 (S1-2 Table). For control, yeast was co-transformed with plasmid combination of UpYES-6xHis-XPO1, LpGAD-CUP-6xHis-p92 and HpGBK-CUP-6xHis-p33/Gal: DI72. The co-purification experiment was performed according to previous description [28, 105]. Briefly, the transformed yeasts were cultured on SC-ULH^-^ plates. Single colony was picked and streaked on SC-ULH^-^ plate followed by inoculation into 20 mL SC-ULH^-^ media containing 2% glucose and 100 μM BCS. After 20 h cultivation at 23 ℃, the yeast pellet was obtained after centrifugation at 5,000 rpm, followed by rinsing with sterile water for 3x times. Then, the yeast cells were transferred into 40 mL SC-ULH^-^ media supplemented with 2% galactose and 100 μM BCS and grew for 24 h at 23 ℃. Then, the yeast cells were transferred into 40 mL SC-ULH^-^ media containing 2% galactose and 50 μM CuSO_4_ and incubated for 6 h at 23 ℃ followed by collecting the cells and centrifugation at 5,000 rpm. The obtained yeast pellets were washed by PBS buffer [106] and aliquoted into new tubes (0.2 g of yeasts). The aliquoted yeast pellets were suspended with 200 μL High Salt TG buffer (50 mM Tris-HCl, pH 7.5, 10% glycerol, 0.5 M NaCl, 15 mM MgCl_2_, 10 mM KCl, 1% (V/V), Yeast Protease Inhibitor Cocktail (YPIC)), and was broken using glass beads in FastPrep Homogenizer (MP Biomedicals). This was followed by 5 min centrifugation at 500 g at 4 ℃. Note that we repeated the homogenization and centrifugation steps additional 4x times to increase yield in supernatant. The pellets containing the membrane fraction of yeast were collected via 20 min centrifugation of the above supernatant at 35,000g at 4 ℃. The membrane fraction was solubilized with High salt TG buffer containing 2% (V/V) Triton X100 (8 h rotation in incubator at 4 ℃), and the supernatant was obtained after 20 min centrifugation at 35,000 g at 4 ℃. Then, the supernatant was loaded onto a Bio-Spin chromatography column (Bio-Rad) containing 20 μL Anti-Flag M2 resin (Sigma), followed by 8 h incubation in a rotation incubator at 4 ℃. Then, the columns were washed with High Salt TG buffer 5x times. 30 μL 1x SDS loading buffer (no β−mercaptoethanol) was added at 85 ℃ for 6 min to obtain the eluant, followed by 5 min centrifugation at 500 g. The eluted samples were then treated with β-mercaptoethanol and boiled for 20 min. The affinity-purified Flag-p33 was detected with anti-flag antibody and co-purified 6xHis-XPO1 was detected with anti-His antibody using NBP-BCIP method in western blot assay [65].

***In vitro* droplet formation assay.** The *in vitro* droplet and co-droplet formation assays were performed as published [65]. We used purified 2.5 μM TBSV His_6_-eGFP-p33C in 40 μL reaction volume containing 30 mM Tris-HCl (pH 7.5), 150 mM NaCl and 2.5% PEG8000 and then were incubated in 384 Well glass bottom plates (Cellvis) at room temperature for 20 min and monitored using an Olympus FV3000 confocal microscope. The co-droplet formation assay was performed as the droplet formation assay above, except additional purified His_6_-mRFP-tagged DRB4 and His_6_-mRFP-CenH3 were included (as shown in S28 Fig).
