# Supplementary material for: Mobilization of nuclear antiviral factors by exportin XPO1 via the actin network inhibits RNA virus replication
Source: PLoS Pathog. 2025 Aug 19;21(8):e1012841. doi: 10.1371/journal.ppat.1012841 (PMC12393752; doi:10.1371/journal.ppat.1012841)
Supplement: S1 Table — (DOCX) [file ppat.1012841.s030.docx]

**S1 Table. List of plasmids constructed during this work**

| **#** | **Plasmid Name** | **Insert source** | **Insert digestion  sites** | **Primers used for insert amplification** | **Vector source** | **Vector digestion  sites** |
| --- | --- | --- | --- | --- | --- | --- |
| 1 | pGD-eGFP-XPO1 | cDNA of *Arabidopsis thaliana* | *BamH* I & *Sal* I | #7221 & #7222 | pGD-N-eGFP-MCS | *BamH* I & *Sal* I |
| 2 | pGD-RFP-XPO1 | cDNA of *A. thaliana* | *BamH* I & *Sal* I | #7221 & #7222 | pGD-N-RFP-MCS | *BamH* I & *Sal* I |
| 3 | pGD-TRV::NbXPO1 | cDNA of *Nicotiana. benthamiana* | *Xba* I & *Xho* I | #8462 & #8463 | pGD-TRV2-MCS | *Xba* I & *Xho* I |
| 4 | pGD-CoxIV-BFP | pGD-CoxIV-RFP | *BamH* I & *Pst* I | #6536 & #6537 | pGD-MCS-C-BFP | BamH I & Pst I |
| 5 | pGD-nYFP-XPO1 | cDNA of *N. benthamiana* | *BamH* I & *Sal* I | #7221 & #7222 | pGD-nYFP-MCS | *BamH* I & *Sal* I |
| 6 | UpYES-NT-6xHis-XPO1 | cDNA of *N. benthamiana* | *BamH* I & *Sal* I | #7221 & #7222 | UpYES-NT-6xHis-MCS | *BamH* I & *Xho* I |
| 7 | pGD-Flag-AGO2 | cDNA of *N. benthamiana* | *BamH* I & *Sal* I | #8439 & #8440 | pGD-N-Flag-MCS | *BamH* I & *Sal* I |
| 8 | pGD-eGFP-AGO2 | cDNA of *N. benthamiana* | *BamH* I & *Sal* I | #8439 & #8440 | pGD-N-eGFP-MCS | *BamH* I & *Sal* I |
| 9 | pGD-Flag-DRB4 | cDNA of *A. thaliana* | *BamH* I & *Sal* I | #8444 & #8445 | pGD-N-Flag-MCS | *BamH* I & *Sal* I |
| 10 | pGD-eGFP-DRB4 | cDNA of *A. thaliana* | *BamH* I & *Sal* I | #8444 & #8445 | pGD-N-eGFP-MCS | *BamH* I & *Sal* I |
| 11 | pGD-nYFP-RanGAP1 | cDNA of *N. benthamiana* | *BamH* I & *Sal* I | #9011 & #9012 | pGD-nYFP-MCS | *BamH* I & *Sal* I |
| 12 | pGD-eGFP-RanGAP1 | cDNA of *N. benthamiana* | *BamH* I & *Sal* I | #9011 & #9012 | pGD-N-eGFP-MCS | *BamH* I & *Sal* I |
| 13 | pGD-nYFP-RanGAP2 | cDNA of *N. benthamiana* | *BamH* I & *Sal* I | #9013 & #9014 | pGD-nYFP-MCS | *BamH* I & *Sal* I |
| 14 | pGD-eGFP-RanGAP2 | cDNA of *N. benthamiana* | *BamH* I & *Sal* I | #9013 & #9014 | pGD-N-eGFP-MCS | *BamH* I & *Sal* I |
| 15 | pGD-nYFP-RanBP1-1b | cDNA of *N. benthamiana* | *BamH* I & *Sal* I | #9015 & #9017 | pGD-nYFP-MCS | *BamH* I & *Sal* I |
| 16 | pGD-eGFP-RanBP1-1b | cDNA of *N. benthamiana* | *BamH* I & *Sal* I | #9015 & #9017 | pGD-N-eGFP-MCS | *BamH* I & *Sal* I |
