# Supplementary material for: Mobilization of nuclear antiviral factors by exportin XPO1 via the actin network inhibits RNA virus replication
Source: PLoS Pathog. 2025 Aug 19;21(8):e1012841. doi: 10.1371/journal.ppat.1012841 (PMC12393752; doi:10.1371/journal.ppat.1012841)
Supplement: S2 Table — (DOC) [file ppat.1012841.s031.doc]

**S2 Table. List of plasmids from prior works**

| **Number** | **Plasmid Name** | **Source (reference)** |
| --- | --- | --- |
| 1 | pGD-EV | [107] |
| 2 | pGD-TRV1 | [108] |
| 3 | pGD-TRV::cGFP | [108] |
| 4 | pGD-TRV::GST | Gift from Dr. Molho |
| 5 | pGD-p33-RFP | [107] |
| 6 | pGD-p33-BFP | [107] |
| 7 | pGD-RFP-SKL | [107] |
| 8 | pGD-p36-RFP | [107] |
| 9 | pGD-p36-BFP | [107] |
| 10 | pGD-CoxIV-RFP | [107] |
| 11 | pGD-p33-cYFP | [107] |
| 12 | pGD-p36-cYFP | [107] |
| 13 | LpGAD-CUP-Flag-p92 | Gift from Dr. Wenwu Lin |
| 14 | HpGBK-CUP-Flag-p33/Gal: DI72 | Gift from Dr. Wenwu Lin |
| 15 | LpGAD-CUP-6xHis-p92 | Gift from Dr. M. Molho |
| 16 | HpGBK-CUP-6xHis-p33/Gal: DI72 | Gift from Dr. M. Molho |
| 17 | pGD-eGFP-CenH3 | [109] |
| 18 | pB7m24GW-pro35S-lifeactmRuby3 | gift from Dr.Tomo Kawashima  (University of Kentucky) |
| 19 | pGD-Flag-RavK | [66] |
| 20 | pGD-Flag-VipA | [66] |
| 21 | pGD-p19 | [107] |
