# Supplementary material for: Mobilization of nuclear antiviral factors by exportin XPO1 via the actin network inhibits RNA virus replication
Source: PLoS Pathog. 2025 Aug 19;21(8):e1012841. doi: 10.1371/journal.ppat.1012841 (PMC12393752; doi:10.1371/journal.ppat.1012841)
Supplement: S3 Table — (DOCX) [file ppat.1012841.s032.docx]

**S3 Table. List of primers used**

| **#** | **Name of the primer** | **Sequence of the primer (5’-3’)** | **Restriction digestion sites** |
| --- | --- | --- | --- |
| #7221 | *BamH* I/AtXPO1-F | CG**ggatcc**ATGGCGGCTGAGAAGTTAAGG | *BamH* I |
| #7222 | AtXPO1-stop/ *Sal* I-F | ACGC**gtcgac**TTATGAGTCCACCATCTCGTC | *Sal* I |
| #8439 | *BamH* I/NbAGO2-F | CGC**ggatcc**ATGGATCGTGGAAATTACCG | *BamH* I |
| #8440 | NbAGO2-stop/*Sal* I -R | ACGC**gtcgac**TCAGACAAAGAACATTATGTTCTGCAGC | *Sal* I |
| #8444 | *BamH* I/AtDRB4-F | CGC**ggatcc**ATGGATCATGTATACAAAGGTCAACT | *BamH* I |
| #8445 | AtDRB4-stop/*Sal* I-R | ACGC**gtcgac**TTATGGCTTCACAAGACGATAGGCTAT | *Sal* I |
| #8462 | TRV::NbXPO1a/*Xba* I-F | GC**tctaga**AGACAACTTTATGCTGGTCC | *Xba* I |
| #8463 | TRV::NbXPO1a*/Xho* I-R | CCG**ctcgag**GATCAATGTTTCCCTCATGA | *Xho* I |
| #8825 | qPCR-F-NbXPO1 | GAGTTCTCCTCTCAGGACAATAAG |  |
| #8826 | qPCR-R-NbXPO2 | AGGTGCAATTAGACCAGGAATAG |  |
| #2859 | qPCR-F-NbTubulin | TAATACGACTCACTATAGGAACCAAATCATTCATGTTGCTCTC |  |
| #2860 | qPCR-R-NbTubulin | TAGTGTATGTGATATCCCACCAA |  |
| #9011 | *BamH* I/NbRanGAP1-F | GCG**ggattc**ATGGATTCTGCAGGATTCTCT | *BamH* I |
| #9012 | NbRanGAP1/*Pst* I/ stop/*Sal* I-R | ACGC**gtcgac**TTA**ctgcag**ATTCTTCCTGCTTGATATCAAGACCCTTG | Pst I and *Sal* I |
| #9013 | *BamH* I/NbRanGAP2-F | CGC**ggatcc**ATGGATGCCACAACGC | *BamH* I |
| #9014 | NbRanGAP2/*Hind* III/ stop/Sal I-R | ACGC**gtcgac**TTA**aagctt**CTTGACGTCAAGGTTTTTGAGTTTTGATTC | *Hind* III and *Sal* I |
| #9015 | *Xba* I/*BamH* I/ NbRanBP1-F | GC**tctagaggatcc**ATGGCAAGCAGTGCAGAGC | *Xba* I and *BamH* I |
| #9017 | NbRanBP1-1b/*Sma* I/ stop/*Sal* I-R | ACGC**gtcgac**TTA**cccggg**ATTCTTCTCATCAGCATTTTCTTCCTTCTTA | *Sma* I and *Sal* I |
